# The All of Us Research Program’s wearables dataset

**DOI:** 10.1038/s41591-026-04352-3

**Published:** 2026-04-27

**Authors:** Theresa Patten, Edward A. Preble, Hiral Master, Jennifer Adjemian, Andrea Ramirez, James McClain, Amy Rose Price

**Affiliations:** 1 National Institutes of Health, Bethesda, MD USA; 2 RTI International, Research Triangle Park, NC USA; 3 Vanderbilt Institute of Clinical and Translational Research, Vanderbilt University Medical Center, Nashville, TN USA; 4 Department of Health Sciences, School of Medicine, University of North Carolina at Chapel Hill, Chapel Hill, NC USA

**Keywords:** Epidemiology, Risk factors

## Abstract

Digital health technologies (DHTs) are revolutionizing medical research, offering unprecedented insights into health monitoring and disease detection through continuous, real-world data collection. Here we characterize the data in one of the largest and most demographically rich DHT datasets as part of the All of Us Research Program. Through a historic device distribution effort, the program reached a broad range of participants nationwide, yielding a DHT dataset with an expanded a large demographic scope. This dataset contains Fitbit data from more than 59,000 participants spanning 14 years with more than 39 million step observations and 31 million sleep observations. Nearly half (46%) of participants with Fitbit data also contributed electronic health records, physical measurements, genomics and survey data. This resource enables researchers to study relationships between digital health metrics and clinical outcomes, advancing DHT methodologies through its large size, broad representation and multi-modal data linkage.

## Abstract (web summary)

The authors present the All of Us Research Program dataset, containing Fitbit data from 59,000 participants spanning 14 years, 39 million steps and 31 million sleep observations.

## Main

DHTs are fundamentally transforming biomedical research by enabling continuous, real-world health monitoring at unprecedented scale and granularity. Commercial wearable devices, now owned by 20–45% of people in the USA^1–3^, generate rich streams of physiological and behavioral data that can inform clinical decision-making and improve patient outcomes^4,5^. However, the potential impact of these technologies remains constrained by substantial demographic biases, with wearables ownership and much of DHT research disproportionately representing white individuals with higher educational and income levels^3,6,7^. This gap in representation limits our understanding of how digital biomarkers manifest across demographic groups and creates barriers to developing health interventions based on DHT data that benefit all.

The National Institutes of Health’s All of Us Research Program is a historic initiative to collect health data from 1 million or more people living in the USA and to make these data available for research purposes to registered users. The program’s mission is to address historical gaps in research experience and advance precision health for all, particularly those from populations with unique life experiences and health needs (for example, older adults, rural populations, individuals with less access to healthcare)^8^.

Since November 2020, the program has made de-identified Fitbit data available to researchers through its Bring Your Own Device (BYOD) program, enabling novel investigations that integrate DHT measures with other rich data types such as electronic health records (EHR), physical measurements and surveys. However, like many DHT datasets, BYOD data lacked sufficient representation from broad demographic populations needed to advance health research and precision medicine^9^. To address this limitation, the All of Us Research Program created the Wearables Enhancing All of Us Research (WEAR) study—a device distribution effort that provided Fitbit devices to invited participants from across the USA at no cost^10^. This strategic approach substantially expanded the breadth of participants contributing wearables data to the All of Us Research Program, including from many populations that have been historically underrepresented in DHT research.

The most recent data release provides registered researchers with Fitbit data from more than 59,000 participants, spanning 14 years and including 39 million step observations and 31 million sleep observations. This Resource paper presents a characterization of the expanded WEAR study dataset, documenting its size and the breadth of participant representation. The combined WEAR and BYOD dataset represents one of the largest wearables datasets available for biomedical research, enabling investigators to examine patterns of physical activity, sleep and their relationship to health outcomes across numerous population groups. By linking wearables data with other rich data types such as genomics, EHRs and survey responses, this resource creates opportunities to advance our understanding of digital biomarkers and their clinical applications. We also provide methodological considerations and frameworks for responsible use to help researchers maximize the scientific and societal impact of this valuable resource.

## Results

### Cohort size and demographics

**Fig. 1:**
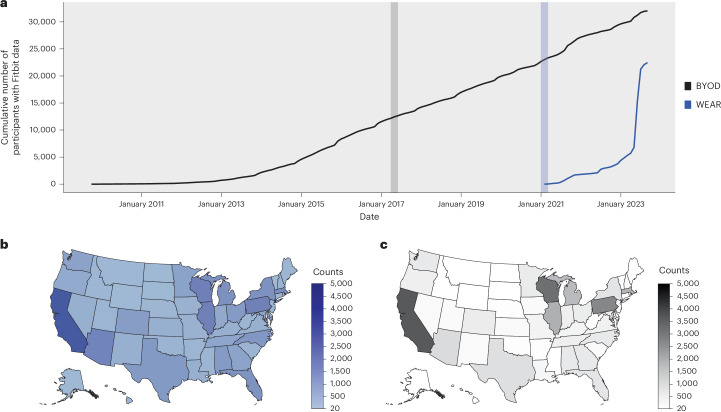
Temporal and geographic distribution of Fitbit data availability. **a**, Cumulative count of participants contributing Fitbit data over time from the BYOD program (black line) and the WEAR study (blue line). Vertical lines indicate the start of enrollment for the All of Us Research Program (gray) and WEAR study (blue). **b**, Geographic distribution of participants in the WEAR program (*n* = 25,072). **c**, Geographic distribution of participants in the BYOD study (*n* = 33,946). US state boundaries in **b** and **c** rendered using Plotly Express (px.choropleth) with the built-in USA-states geometry. Plotly’s geographic boundaries are open source and distributed as part of the Plotly library, with equivalent GeoJSON files publicly hosted in Plotly’s datasets repository (https://github.com/plotly/datasets).

Enrollment in the All of Us Research Program and the WEAR study began in May 2017 and February 2021, respectively, as indicated by gray and blue vertical lines in Fig. 1a. The number of participants contributing data through BYOD has grown steadily and includes data from before enrollment began for the All of Us Research Program. This is possible because participants can donate all historical Fitbit data when they consent, including activity data recorded before they enrolled in the program. The WEAR study began as a limited pilot in 2021, with enrollment increasing gradually through protocol refinements in 2022. A major expansion from April to June 2023 broadened the eligibility criteria and initiated large-scale participant recruitment (Fig. 1a). Both BYOD and WEAR programs show similar and widespread participation across the country, with participants from all 50 states (Fig. 1b,c). Geographic distributions show similar patterns between BYOD and WEAR, with the highest concentrations in states such as California, Wisconsin, Pennsylvania and Illinois where the All of Us Research Program has large healthcare provider organization partners.

To measure the success of the WEAR study in collecting Fitbit data from participants with varying health needs and research experiences, we compared the demographic characteristics of those who donated activity data through the BYOD program (*n* = 32,035) to WEAR study participants (*n* = 22,474) in our general activity analysis cohort (Table 1). As expected, the demographic profile of the WEAR study cohort differed from the BYOD cohort across a range of characteristics, including self-reported race and ethnicity (for example, 77.3% versus 55.1% of participants reported being white), age (for example, 22.3% versus 15.7% of participants reported an age between 55 and 64 years), income (for example, 6.1% versus 15.2% of participants reported a household annual income between US$10,000 and US$25,000), education (for example, 32.2% versus 27.2% of participants reported having a college degree), healthcare access and utilization (32.8% versus 43.4% of participants reported inadequate access to healthcare) and disability status (for example, 1.8% versus 4.1% reported blindness or difficulty seeing) (Table 1).

**Table 1:** Demographic characteristics of general activity cohort BYOD and WEAR participants

Variable	BYOD	WEAR	Total
	*n* (%)	*n* (%)	*n* (%)
	32,035 (58.8)	22,474 (41.2)	54,509
**Race** **or** **ethnicity**			
White	24,749 (77.3)	12,380 (55.1)	37,129 (68.1)
Black	1,768 (5.5)	2,174 (9.7)	3,942 (7.2)
Hispanic or Latino	1,415 (4.4)	2,442 (10.9)	3,857 (7.1)
Asian	1,040 (3.2)	1,447 (6.4)	2,487 (4.6)
Middle Eastern or North African	105 (0.3)	203 (0.9)	308 (0.6)
American Indian or Alaska Native	62 (0.2)	130 (0.6)	192 (0.4)
Native Hawaiian or Other Pacific Islander	<20 (0.1)	<20 (0.1)	25 (0.0)
Generalized multiple populations	2,362 (7.4)	3,089 (13.7)	5,451 (10.0)
None of these	236 (0.7)	323 (1.4)	559 (1.0)
Skip	241 (0.8)	229 (1.0)	470 (0.9)
Prefer not to answer	<60 (0.2)	<60 (0.3)	89 (0.2)
**Age (years)**			
Mean	48.9	53.1	50.6
Median [minimum, maximum]	50.0 [18.0, 109]	54.0 [18.0, 97.0]	51.0 [18.0, 109]
**Age group**			
18–24	1,879 (5.9)	999 (4.4)	2,878 (5.3)
25–34	5,356 (16.7)	3,227 (14.4)	8,583 (15.7)
35–44	5,679 (17.7)	3,694 (16.4)	9,373 (17.2)
45–54	6,052 (18.9)	3,341 (14.9)	9,393 (17.2)
55–64	7,146 (22.3)	3,531 (15.7)	10,677 (19.6)
65–74	4,981 (15.5)	5,357 (23.8)	10,338 (19.0)
75–84	908 (2.8)	2,160 (9.6)	3,068 (5.6)
85+	34 (0.1)	165 (0.7)	199 (0.4)
**Sex at birth**			
Female	22,702 (70.9)	14,459 (64.3)	37,161 (68.2)
Male	9,216 (28.8)	7,918 (35.2)	17,134 (31.4)
Unknown	>80 (0.2)	>80 (0.4)	>160 (0.3)
Intersex	<20 (0.1)	<20 (0.1)	<40 (0.1)
Other	<20 (0.1)	<20 (0.1)	<40 (0.1)
**Household annual income** **(US$)**			
Less than 10,000	937 (2.9)	1,830 (8.1)	2,767 (5.1)
10,000–24,999	1,961 (6.1)	3,423 (15.2)	5,384 (9.9)
25,000–34,999	1,912 (6.0)	1,932 (8.6)	3,844 (7.1)
35,000–49,999	2,933 (9.2)	2,402 (10.7)	5,335 (9.8)
50,000–74,999	5,065 (15.8)	3,313 (14.7)	8,378 (15.4)
75,000–99,999	4,605 (14.4)	2,553 (11.4)	7,158 (13.1)
100,000–149,999	6,315 (19.7)	2,900 (12.9)	9,215 (16.9)
150,000–199,999	2,990 (9.3)	1,199 (5.3)	4,189 (7.7)
More than 200,000	3,519 (11.0)	1,258 (5.6)	4,777 (8.8)
Unknown	1,798 (5.6)	1,664 (7.4)	3,462 (6.4)
**Education**			
Never attended	<20 (0.1)	<20 (0.1)	<40 (0.1)
Grades 1–4	<20 (0.1)	<20 (0.1)	<40 (0.1)
Grades 5–8	>20 (0.1)	>50 (0.2)	>70 (0.1)
Grades 9–11	159 (0.5)	416 (1.9)	575 (1.1)
Grade 12 or GED	1,975 (6.2)	2,365 (10.5)	4,340 (8.0)
College 1–3 years	7,624 (23.8)	6,854 (30.5)	14,478 (26.6)
College graduate	10,305 (32.2)	6,105 (27.2)	16,410 (30.1)
Advanced degree	11,699 (36.5)	6,466 (28.8)	18,165 (33.3)
Unknown	239 (0.7)	169 (0.8)	408 (0.7)
**Healthcare access and utilization**			
Adequate access to healthcare	21,535 (67.2)	12,713 (56.6)	34,248 (62.8)
Inadequate access to healthcare	10,500 (32.8)	9,761 (43.4)	20,261 (37.2)
**Disability**			
Are you blind or do you have serious difficulty seeing, even when wearing glasses?			
Yes	516 (1.8)	889 (4.1)	1,405 (2.8)
No	27,887 (98.2)	20,891 (95.9)	48,778 (97.2)
Are you deaf or do you have serious difficulty hearing?			
Yes	1,582 (5.6)	2,055 (9.4)	3,637 (7.2)
No	26,855 (94.4)	19,739 (90.6)	46,594 (92.8)
Because of a physical, mental or emotional condition, do you have serious difficulty concentrating, remembering or making decisions?			
Yes	3,188 (11.3)	4,196 (19.5)	7,384 (14.8)
No	25,027 (88.7)	17,363 (80.5)	42,390 (85.2)
Do you have serious difficulty walking or climbing stairs?			
Yes	2,179 (7.7)	3,431 (15.8)	5,610 (11.2)
No	26,217 (92.3)	18,292 (84.2)	44,509 (88.8)
Do you have difficulty dressing or bathing?			
Yes	636 (2.2)	1,090 (5.0)	1,726 (3.4)
No	27,766 (97.8)	20,655 (95.0)	48,421 (96.6)
Because of a physical, mental or emotional condition, do you have difficulty doing errands alone such as visiting the doctor’s office or shopping?			
Yes	1,621 (5.7)	2,430 (11.2)	4,051 (8.1)
No	26,798 (94.3)	19,294 (88.8)	46,092 (91.9)

Some counts are suppressed in accordance with the All of Us Data and Statistics Dissemination Policy to prevent reporting or potential triangulation of small cell sizes (*n* < 20). GED, general education development.

### Cohort-level trends in daily steps and sleep duration

We quantified high-level wearables metrics in our general activity cohort (*n* = 54,509) and general sleep cohort (*n* = 34,378). Participants in these cohorts recorded a median of 6,454 daily steps (interquartile range (IQR) 4,432–8,958) and a median daily sleep duration of 6.8 h (IQR 6.2–7.2), respectively (Table 2; ‘Full cohort’). In addition, using the sleep duration categories defined previously^11^, we found that 36.6% (*n* = 12,592) of participants in our general sleep cohort had median sleep durations in the normal range (7–9 h per night), while 61.1% (*n* = 21,020) had short sleep (5–7 h per night), 1.4% (*n* = 492) had very short sleep (<5 h per night) and 0.8% (*n* = 274) had long sleep (≥9 h per night)^12^.

**Table 2:** Baseline wearables outcomes by cohort with select demographic group comparisons

	Median no. of daily steps (*n*; IQR)			Median daily sleep duration (h) (*n*; IQR)		
	BYOD	WEAR	General activity cohort	BYOD	WEAR	General sleep cohort
Full cohort	6,867 (*n* = 32,035; 4,929– 9,337)	5,797 (*n* = 22,474; 3,772–8,336)	6,454 (*n* = 54,509; 4,432–8,958)	6.8 (*n* = 21,794; 6.3–7.3)	6.6 (*n* = 12,584; 6.1–7.2)	6.8 (*n* = 34,378; 6.2–7.2)
**Sex at birth**						
Female	6,564 (*n* = 22,702; 4,721–8,886)	5,355 (*n* = 14,459; 3,528–7,698)	6,114 (*n* = 37,161; 4,225–8,442)	6.9 (*n* = 15,412; 6.3–7.3)	6.7 (*n* = 7,905; 6.1–7.3)	6.8 (*n* = 23,317; 6.3–7.3)
Male	7,719 (*n* = 9,216; 5,544–10,270)	6,746 (*n* = 7,918; 4,403–9,581)	7,264 (*n* = 17,134; 5,046–10,001)	6.6 (*n* = 6,302; 6.1–7.1)	6.5 (*n* = 4,623; 6.0–7.1)	6.6 (*n* = 10,925; 6.0–7.1)
Intersex	<20	<20	<40	<20	<20	<40
Other	<20	<20	<40	<20	<20	<40
Unknown	>80	>50	>130	>50	>30	>80
**Age category**						
18–24	7,150 (*n* = 1,879; 5,375–9,084)	6,509 (*n* = 999; 4,575–8,819)	6,948 (*n* = 2,878; 5,112–9,017)	7.0 (*n* = 1,502; 6.5–7.4)	6.7 (*n* = 553; 6.2–7.3)	7.0 (*n* = 2,055; 6.4–7.3)
25–34	6,954 (*n* = 5,356; 5,211–9,038)	6,272 (*n* = 3,227; 4,418–8,464)	6,732 (*n* = 8,583; 4,908–8,848)	6.9 (*n* = 4,074; 6.4–7.3)	6.7 (*n* = 1,820; 6.1–7.3)	6.8 (*n* = 5,894; 6.3–7.3)
35–44	6,861 (*n* = 5,679; 5,000 –9,331)	6,068 (*n* = 3,694; 4,044–8,525)	6,564 (*n* = 9,373; 4,582–9,045)	6.8 (*n* = 3,778; 6.2–7.2)	6.6 (*n* = 1,896; 6.0–7.1)	6.7 (*n* = 5,674; 6.2–7.2)
45–54	6,906 (*n* = 6,052; 4,993–9,305)	5,603 (*n* = 3,341; 3,709–8,078)	6,500 (*n* = 9,393; 4,500–8,955)	6.7 (*n* = 4,064; 6.2–7.2)	6.5 (*n* = 1,687; 5.9–7.0)	6.6 (*n* = 5,751; 6.1–7.1)
55–64	7,140 (*n* = 7,146; 5,020–9,919)	5,480 (*n* = 3,531; 3,540–8,278)	6,608 (*n* = 10,677; 4,448–9,460)	6.8 (*n* = 4,722; 6.2–7.2)	6.5 (*n* = 1,834; 5.9–7.1)	6.7 (*n* = 6,556; 6.1–7.2)
65–74	6,484 (*n* = 4,981; 4,460–9,296)	5,856 (*n* = 5,357; 3,660–8,519)	6,169 (*n* = 10,338; 4,061–8,908)	6.8 (*n* = 3,089; 6.3–7.3)	6.8 (*n* = 3,119; 6.2–7.3)	6.8 (*n* = 6,208; 6.2–7.3)
75–84	5,249 (*n* = 908; 3,595–7,645)	5,034 (*n* = 2,160; 3,099–7,376)	5,098 (*n* = 3,068; 3,225–7,427)	6.9 (*n* = 539; 6.3–7.4)	6.8 (*n* = 1,553; 6.2–7.3)	6.8 (*n* = 2,092; 6.2–7.3)
85+	4,638 (*n* = 34; 2,790–6,736)	3,080 (*n* = 165; 1,670–5,135)	3,460 (*n* = 199; 1,774–5,353)	7.1 (*n* = 26; 6.8–7.5)	6.8 (*n* = 122; 6.1–7.4)	6.8 (*n* = 148; 6.2–7.4)
**Race** **or** **ethnicity**						
White	6,898 (*n* = 24,749; 4,935–9,416)	5,413 (*n* = 12,380; 3,380–7,934)	6,434 (*n* = 37,129; 4,369–8,984)	6.9 (*n* = 17,109; 6.3–7.3)	6.8 (*n* = 6,779; 6.2–7.3)	6.8 (*n* = 23,888; 6.3–7.3)
American Indian or Alaska Native	5,598 (*n* = 62; 3,487–8,508)	5,695 (*n* = 130; 3,503–9,163)	5,658 (*n* = 192; 3,490–8,589)	6.5 (*n* = 35; 6.0–7.0)	6.4 (*n* = 52; 5.6–6.9)	6.4 (*n* = 87; 5.7–7.0)
Asian	7,490 (*n* = 1,040; 5,712–9,920)	7,038 (*n* = 1,447; 5,174–9,512)	7,289 (*n* = 2,487; 5,386–9,693)	6.5 (*n* = 722; 6.0–6.9)	6.4 (*n* = 924; 5.9–6.9)	6.4 (*n* = 1,646; 6.0–6.9)
Black	6,346 (*n* = 1,768; 4,544–8,603)	5,538 (*n* = 2,174; 3,836–8,004)	5,933 (*n* = 3,942; 4,147–8,329)	6.2 (*n* = 1,029; 5.7–6.7)	6.3 (*n* = 1,142; 5.7–6.8)	6.2 (*n* = 2,171; 5.7–6.8)
Hispanic or Latino	7,178 (*n* = 1,415; 5,276–9,493)	6,733 (*n* = 2,442; 4,650–9,235)	6,899 (*n* = 3,857; 4,929–9,323)	6.6 (*n* = 933; 6.1–7.1)	6.5 (*n* = 1,446; 6.0–7.0)	6.6 (*n* = 2,379; 6.0–7.1)
Middle Eastern or North African	7,429 (*n* = 105; 5,525–9,346)	6,599 (*n* = 203; 4,540–8,984)	6,800 (*n* = 308; 4,741–9,140)	6.7 (*n* = 78; 6.4–7.1)	6.6 (*n* = 142; 6.1–7.1)	6.7 (*n* = 220; 6.2–7.1)
Native Hawaiian or Other Pacific Islander	<20	<20	<40	<20	<20	<40
Generalized multiple populations	6,546 (*n* = 2,362; 4,704–8,928)	6,081 (*n* = 3,089; 4,044–8,462)	6,300 (*n* = 5,451; 4,334–8,684)	6.8 (*n* = 1,566; 6.2–7.2)	6.6 (*n* = 1,767; 6.0–7.2)	6.7 (*n* = 3,333; 6.1–7.2)
None of these	6,391 (*n* = 236; 4,499–8,760)	5,862 (*n* = 323; 4,006–8,612)	6,100 (*n* = 559; 4,231–8,734)	6.8 (*n* = 160; 6.2–7.2)	6.7 (*n* = 198; 5.9–7.1)	6.8 (*n* = 358; 6.0–7.2)
Prefer not to answer	<50	<50	<100	<50	<50	<100
Skip	6,682 (*n* = 241; 4,777–9,316)	5,914 (*n* = 229; 3,595–8,718)	6,309 (*n* = 470; 4,156–9,059)	7.0 (*n* = 130; 6.4–7.4)	6.8 (*n* = 112; 6.1–7.3)	6.9 (*n* = 242; 6.3–7.4)

Some counts are suppressed in accordance with the All of Us Data and Statistics Dissemination Policy to prevent reporting or potential triangulation of small cell sizes (*n* < 20).

Next, we compared high-level wearables metrics between the BYOD and WEAR cohorts, and reported median daily steps and sleep durations across select demographic characteristics, including sex, age, race, ethnicity, and American Indian and Alaska Native status (Table 2). Both median daily steps and sleep duration were significantly higher in the BYOD cohort than in the WEAR cohort (Table 2). Specifically, the BYOD activity cohort recorded a median of 6,867 steps (*n* = 32,035) compared to 5,797 steps in the WEAR activity cohort (*n* = 22,474), and a median sleep duration of 6.8 h in the BYOD sleep cohort (*n* = 21,794) compared to 6.6 h in the WEAR sleep cohort (*n* = 12,584) (Mann–Whitney *U* tests; *P* < 2.2 × 10^−16^ for both comparisons). Stratified comparisons by additional demographic subgroups (for example, sex, age, self-reported race or ethnicity) were not conducted.

### Seasonal trends in daily steps and sleep duration

**Fig. 2:**
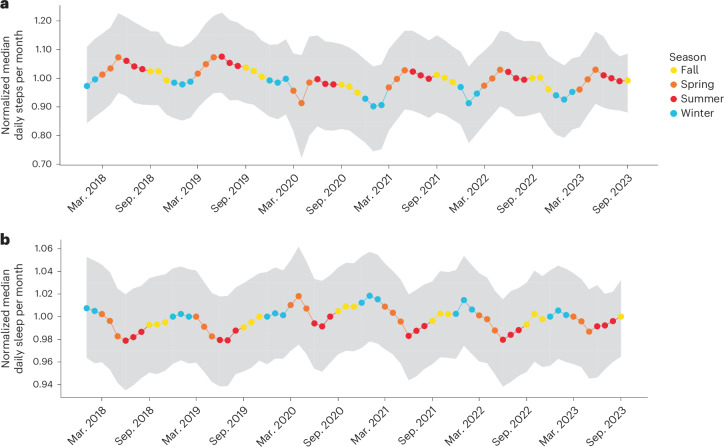
Seasonal variation in physical activity and sleep. **a**, Seasonal variation in normalized median daily steps of All of Us Research Program participants in the seasonal activity cohort (*n* = 53,295). Each participant’s monthly median daily steps were normalized to their overall median daily step count over the entire observation period (3 October 2009 to 30 September 2023). **b**, Seasonal variation in normalized median daily sleep duration of All of Us Research Program participants in the seasonal sleep cohort (*n* = 33,471). Each participant’s monthly median sleep durations were normalized to their overall median sleep duration over the entire observation period (6 October 2009 to 30 September 2023). For both **a** and **b**, normalized data from 1 January 2018 to 30 September 2023 are shown and the shaded area represents the IQR of normalized median daily steps or sleep duration for each month, respectively.

Next, to evaluate whether the All of Us Research Program’s Fitbit dataset reflects established seasonal patterns in physical activity and sleep^13,14^, we calculated the normalized median (IQR) daily steps and sleep durations of eligible participants (per month) and plotted values between January 2018 and September 2023 in Fig. 2 (absolute values are available in Supplementary Fig. 1).

We observed expected seasonal trends in physical activity and sleep, with more steps generally taken in spring and summer than in winter (Fig. 2a; *n* = 53,295) and longer sleep durations in winter (Fig. 2b; *n* = 33,471). However, seasonal variation was more pronounced for steps than sleep (approximately ±10% change versus approximately ±2%) (Fig. 2a,b). A notable exception in both trends occurred in 2020, likely due to the COVID-19 pandemic and associated lockdowns, as has been previously reported^15^. During this year, daily steps continued declining through March and April, while daily sleep durations increased, contrary to typical seasonal patterns.

### Overlap of Fitbit data with other data types

**Fig. 3:**
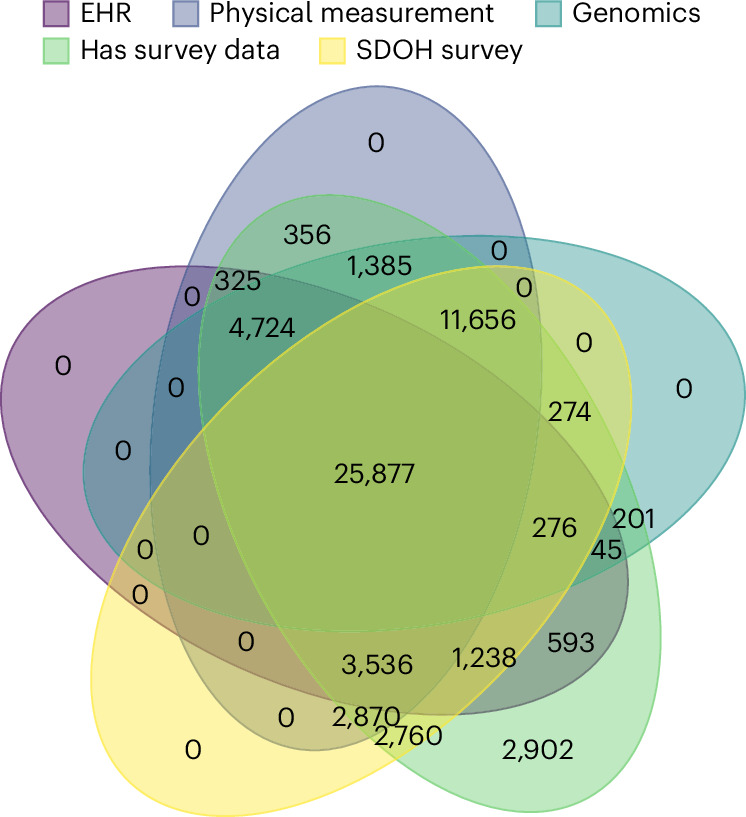
Available data types for those with Fitbit data. Venn diagram displaying the counts of participants with Fitbit data who have EHR, physical measurements, genomics, responses to at least one of the Basics, Family History, Lifestyle, Personal Medical History, Overall Health, and Healthcare Access and Utilization surveys, and responses to the SDOH survey available in the CDR v.8 dataset.

A strength of the All of Us dataset is that it allows registered researchers to bring together many different data types in a secure cloud-computing environment. This report aims to highlight scientific opportunities available using the program’s extensive and demographically rich DHT dataset, rather than pursue novel discoveries. As such, the research questions and analyses described here remain purposefully high level. However, we expect external researchers will combine multiple data types to discover novel associations and risk factors. To highlight the potential power of the DHT dataset to address more complex research questions, we quantified the number of participants who donated multiple data types (Fig. 3). Of the participants who shared Fitbit data, 44% (25,877) also donated EHR data, physical measurements, genomics, responses to at least one of the Basics, Family History, Lifestyle, Personal Medical History, Overall Health, and Healthcare Access and Utilization surveys, and responses to a survey on social determinants of health (SDOH). Incorporating the environmental and social information captured by these surveys into DHT research is essential to ensure impactful advancements in the field using this expanded data resource^16^.

### Case study with wearable and EHR data: recovery in daily steps following lower limb injury

**Fig. 4:**
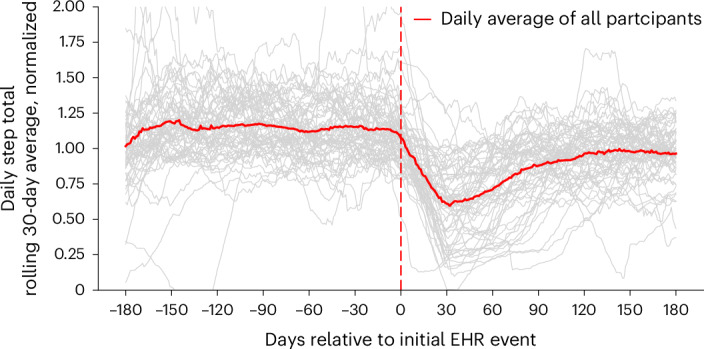
Recovery of daily steps following lower limb fracture. The 30-day rolling averages of normalized daily step counts show an immediate decline from baseline after injury followed by a gradual recovery to near pre-injury levels by 180 days after injury. *n* = 61.

To demonstrate the power of integrating wearables data with another data type in the same individuals, we conducted a case study examining the impact of lower limb fracture on daily step counts. Among 61 participants who sustained a lower limb fracture, the 30-day rolling average of normalized daily steps shows a sharp decline from baseline immediately after injury. This decline continued for ~33 days after injury, dropping to 40% below baseline before gradually recovering to near pre-injury levels by 120 days after injury (Fig. 4).

## Discussion

In this Resource paper, we highlight the value of the All of Us Research Program’s expanded wearables dataset. We examined how multiple DHT outcomes aligned with expected trends previously published in the literature. Specifically, we calculated baseline cohort activity and sleep outcomes in large cohorts of more than 30,000 participants, observed seasonal variations in physical activity and sleep, and presented a case study of the activity trajectory of participants following a lower limb fracture. Together, these analyses demonstrate the value and unique nature of the All of Us Fitbit data resource in terms of its scale, longitudinal observation period and integration with clinical outcomes, including those recorded in the EHR data.

The longitudinal nature of this dataset enables examination of temporal patterns. Although variation in seasonal activity and sleep is relatively well-established, few studies have measured oscillations directly via continuous activity monitoring over several years^13^. An advantage of commercial wearable device data (for example, Fitbit) in large cohort studies is potentially higher compliance and more continuous data.

Analysis of the All of Us dataset revealed expected seasonal variation in physical activity, as measured by median daily steps, including a deviation from this pattern in 2020 owing to the COVID-19 pandemic. This deviation was observed in an earlier analysis of this dataset, but at that time, the sample size was much smaller (*n* = 5,443) and less demographically varied^15^. Interestingly, our analysis shows that median daily steps never fully recovered to pre-pandemic levels (Fig. 2a, years 2021–2023). This incomplete recovery likely reflects two factors. First, 2021 marks the first year that WEAR participants’ step data was incorporated into the seasonal average. As shown in Table 2, WEAR participants have significantly lower step counts than their BYOD counterparts, suggesting this compositional shift in the All of Us cohort contributed to lower step counts beginning in 2021. Second, lingering pandemic-related behavioral changes, such as extended remote work policies, may have also reduced baseline activity. Future work is needed to disentangle these contributing factors. This expanded dataset will strengthen researchers’ ability to study typical physical activity patterns and the factors that influence deviations from these patterns^17^.

We also observed seasonal variation in sleep duration, consistent with other self-reported and objective measures in the literature, which generally show longer sleep in winter and shorter sleep in spring and summer^18–21^. Notably, we observed increased sleep durations starting in winter 2020 that gradually returned to baseline by winter 2023 (Fig. 2b). Self-reported data have documented similar increases in sleep duration during this period^22,23^, as have several studies using objective measures of sleep early in the COVID-19 pandemic^24^. Our data provide additional confirmation of this pattern and extend the observation period through winter 2023, demonstrating a gradual return to baseline.

We observed a median baseline of 6,454 steps per day in our general activity cohort (Table 2). Published estimates from comparable cohorts (for example, UK Biobank and National Health and Nutrition Examination Survey (NHANES)) often report ~9,000–9,600 steps per day^25–27^. However, these comparisons are sensitive to the step-count algorithm utilized^28^.

In addition, All of Us ingests wearable data via the Fitbit Application Programming Interface, which provides summary tables and metrics derived from Fitbit’s proprietary algorithms. As a result, raw accelerometry data are not available to researchers. Although this standardization may improve comparability in All of Us studies, it complicates comparisons with other cohorts that do provide raw accelerometry data (for example, UK Biobank, NHANES). Furthermore, whereas NHANES and UK Biobank distribute devices for 1 week, All of Us participants donated data for extended periods. Finally, All of Us is a broad convenience sample and is not representative of the US population. Despite the WEAR study’s success in increasing the number of people from certain demographic groups (for example, lower income, less access to healthcare), the All of Us dataset is still older, more female and more highly educated than the general US population. Researchers making detailed comparisons to other cohorts or the general population should apply post-stratification or weighting methods to account for sampling and demographic differences.

The recommended daily sleep duration for adults is 7–9 h and self-reported estimates from US adults typically range from 6.5 to 7.5 h^29,30^. We were interested in how these subjective sleep durations, from surveys like the NHANES would compare to the objectively measured sleep durations in our cohort. In our cohort, the median (IQR) daily sleep duration was 6.8 h (6.2–7.2) (Table 2), which is comparable to the NHANES estimates. However, whereas NHANES data suggests that ~32% of US adults experience ‘short sleep’ (<7 h), we found a much larger percentage (62.5%, *n* = 21,612) of participants with a median main sleep duration classified as short or very short sleep (<7 h). Although these differences are interesting to note, our cohort is not nationally representative and uses device-measured rather than self-reported sleep, complicating direct comparisons. In addition, while research suggests self-reported sleep can lead to overestimations^31^, the magnitude of the difference (32% versus 62.5%) suggests additional factors may be involved.

Recent studies of large cohorts using consumer sleep trackers have generated estimates of global sleep patterns, perhaps providing more appropriate comparisons to our device-measured data. One such study reported a slightly longer average sleep duration in its US subset^32^: 6.9 h versus 6.8 h in our cohort. That study measured sleep in ~50,000 Oura ring users who donated an average of ~242 nights of data from January 2021 to January 2022. By contrast, participants in our cohort donated a median of 159 nights of valid sleep data over a median data donation window of 464 days, spanning from 2009 to 2023 (Supplementary Table 1). The cohorts were similar in age and sex, but socioeconomic status—known to influence sleep duration^33^—was not reported^32^. The WEAR program successfully enrolled individuals from lower socioeconomic statuses who are less likely to be included in wearables datasets that rely on independent device purchases (Table 1). The likely difference in socioeconomic composition between the studies may partially explain the lower sleep durations we observed.

Another study using an under-mattress sleep device, the Withings Sleep Analyzer, reported a significantly higher average sleep duration of ~7.5 h for US device users^34^. Validation studies suggest that the Withings device significantly overestimates sleep duration when compared to polysomnography and may do so to a greater extent than Fitbit devices^35,36^. In addition, the Withings Sleep Analyzer study assessed sleep over 9 months in adults who registered to use the device between July 2020 and March 2021, a period that overlapped significantly with the COVID-19 pandemic. Several reports suggest population-level sleep abnormalities during this time, including increased time in bed and total sleep duration^23,37^. Although our dataset includes this pandemic period, it also includes data from many years before and after, which would have mitigated the impact of pandemic-related changes on our longitudinal median sleep duration.

A key strength of the All of Us data is the ability to examine individual-level changes in relation to clinical events. To demonstrate the value of integrating wearable outcomes with clinical events documented in EHR records, we examined daily step counts in participants who experienced a lower limb fracture. Among the 61 participants in this case study, we observed considerable variability in average daily steps both before and after injury. Nevertheless, the cohort showed a rapid decline in steps relative to baseline immediately following the injury, with recovery taking on average 90 days after injury (seen as 120 rolling average days in Fig. 4). Even at 180 days (6 months) after injury, the cohort had not fully returned to pre-injury activity levels. Given the range of injury severity represented in the ‘Fracture of Lower Limb’ concept ID (Supplementary Table 2) and published reports indicating that several of these injuries require recovery times exceeding 6 months—particularly in older adults—this incomplete recovery was expected^38,39^. Our primary purpose in conducting this case study was to demonstrate how wearable data can be integrated with clinical outcomes to understand correlations between health events and changes in activity patterns. Although we chose a relatively straightforward case study with an expected result, future researchers can leverage these integrated data types to identify novel biomarkers and associations between health, physical activity and sleep outcomes.

Realizing the full potential of this dataset requires continued methodological advancement in several areas. For example, the impact of device type on wearable outcomes remains poorly understood, and there are currently no consensus methods for addressing the use of multiple Fitbit devices in a single study or by a single participant^40^. Similarly, approaches for handling missing data in DHT datasets are not standardized. Missing data in these datasets are unlikely to be random and may reflect conscious or subconscious decisions to remove a device, which can correlate with participant characteristics or health states (for example, mood) and introduce bias^41^.

Although Fitbits have demonstrated reasonable reliability compared to gold-standard devices for certain activity and sleep metrics^42–44^, their reliability varies across specific measures (for example, sleep stages, heart rate), populations and device types^40,45,46^. For example, research suggests that Fitbits measure heart rate less reliably in people with darker skin tones because of differences in how sensors optically measure light absorption^40^. In addition, Fitbit step estimation accuracy may be reduced in people with irregular gait patterns from neurological conditions such as Parkinson’s disease, with inaccuracies varying by device type^47^. The effect of these limitations on study findings will depend on the specific research question, outcome measures and population being studied. Researchers should carefully consider these device-specific and population-specific reliability limitations when designing analyses and interpreting results from this dataset.

Another important consideration is that wearables data may be subject to measurement reactivity, where participants temporarily alter their behavior when first provided with activity and sleep trackers. However, the duration of this effect is likely short-lived and depends on the health-related behavior of interest (for example, daily steps versus exercise minutes)^48^. Researchers should consider their research question and observation period carefully and may wish to exclude the first few days or weeks of data donated by participants to avoid bias^49,50^. Given the large-scale and longitudinal nature of the analyses in this manuscript, we chose not to exclude any days of data.

Future research using the All of Us Fitbit dataset will benefit from methodological advancements that address current limitations; however, developing such approaches was beyond the scope of this paper. Instead, our goal was to present the dataset at a high level, with the expectation that the broader research community will leverage it for methodological developments. Encouragingly, the research community has already begun this work, including several reports that specifically evaluate and provide considerations for using the All of Us Fitbit dataset^40,50,51^. Future All of Us wearables data users are encouraged to reference the program’s user support hub (https://support.researchallofus.org), which contains additional information and guidance, including multiple ‘featured workspaces’ with example code and support articles, such as one titled ‘Considerations while using Fitbit data in the All of Us Research Program’.

Finally, analyses of demographic variables and DHT outcomes (for example, daily steps and sleep duration) require careful consideration to avoid misleading conclusions. A strength of the All of Us dataset is that it integrates many data types, including EHR, genomics and extensive self-reported survey data. Specifically, 82% (48,487 out of 59,018) of participants with Fitbit data also responded to the program’s SDOH survey, which asks about social factors like neighborhood, social life and perceived stress. We urge researchers to plan their analyses carefully, consult experts and community members in their research design, and consider all the data the program collects to study factors underlying sleep and activity differences.

An important consideration for all real-world datasets, including the data presented here, is that many factors of data collection are beyond experimenter control, and some of these uncontrolled factors may introduce sources of error or bias. For example, participants in our cohort used 41 different Fitbit device models with various sensors and technologies (Supplementary Table 3). This device heterogeneity may affect measurement accuracy owing to device-specific limitations or user-selected settings. In addition, although 59,018 participants donated Fitbit data to All of Us, only 52,860 (89.6%) had device information available in the device table, and a small fraction of participants showed evidence of using five or more devices during their data donation window (Supplementary Fig. 2). Such data characteristics reflect the real-world nature of this dataset, in which participants use their own devices over extended periods under free-living conditions.

Because our goal was to provide a high-level overview of Fitbit data availability and trends as a Resource paper, and because there are currently no consensus methods in the field for addressing device heterogeneity in consumer-grade wearables research, we did not prescribe specific analytical approaches for handling these factors. Establishing such methods is an active area of research that extends beyond the scope of a resource description paper. As the field continues to evolve, researchers should carefully consider potential sources of error or bias when analyzing real-world data, and important findings obtained in observational real-world datasets should ideally be followed up with controlled interventional studies when feasible.

In sum, although potential errors and biases present challenges when working with real-world data, there are also crucial benefits that make real-world datasets a valuable resource for the research community. These include their massive scale, richness of longitudinal data, integration with multiple data types (for example, EHR, surveys, genomics), and ability to support a wide range of research objectives—benefits that are often difficult to obtain in more controlled, small-scale datasets. The All of Us Fitbit dataset, with its extended observation periods, large and diverse participant population, and linkage to clinical outcomes, offers opportunities for discovery that complement findings from traditional research-grade accelerometry and plethysmography studies.

The WEAR study was a strategic and innovative effort by the All of Us Research Program to expand the number and representativeness of individuals donating DHT data by distributing Fitbit devices to participants at no cost. WEAR’s success is evidenced by a larger proportion of participants from varying backgrounds donating activity data through the WEAR study relative to the BYOD program (Table 1). The All of Us Research Program is accelerating research in precision medicine, a field that initially focused on the potential for human genetics to enable individually tailored treatments and improve health outcomes, but that over time has broadened its scope to appreciate the role of additional data types, including DHT. By substantially increasing the amount of DHT available from a broader range of individuals across the US population, the expanded All of Us Fitbit dataset offers a valuable resource to advance biomedical research. This resource can help researchers better understand the contributions of sleep, heart rate and physical activity on important health outcomes, and inform the development of more precise treatments and interventions.

## Methods

This research complies with all relevant ethical regulations. Specifically, secondary use of All of Us Research Program data has been designated nonhuman participants research by the All of Us Institutional Review Board. Therefore, additional informed consent was not required. In addition, this study followed the Strengthening the Reporting of Observational Studies in Epidemiology (STROBE) guideline for cohort studies^52^.

### Data sources and participants

Using the Controlled Tier CDR v.8 (C2024Q3R5) on the All of Us Researcher Workbench, we analyzed demographic and Fitbit data from participants who live in the USA or its territories and were aged 18 or older who enrolled in the All of Us Research Program either at a healthcare provider site or by directly visiting the enrollment website between 31 May 2017 and 1 October 2023. There are two pathways by which a participant could donate Fitbit data to the All of Us Research Program: the BYOD program^53^, in which a participant consents to share data from an existing device, or the WEAR study, in which participants were provided with a Fitbit device by the program at no cost. WEAR participants were given a choice between the Fitbit Charge and the Fitbit Versa. Over the course of the study, a variety of each model (for example, Charge 3, Charge 4) was distributed to WEAR participants. WEAR study participants in this data release enrolled between February 2021 and September 2023. We classified participants as WEAR if they consented to join the WEAR program and had data starting on or after 22 February 2021. Participants without WEAR consent or who began donating Fitbit data before 22 February 2021 were considered BYOD participants. Demographic details of all participants were obtained from the All of Us ‘the Basics’ survey.

C2024Q3R5 includes data from 59,018 participants who contributed Fitbit data spanning over 14 years, with data types such as daily and intraday or sequence level metrics for activity, steps, heart rate, sleep and device information (Supplementary Table 1). A subset of this Fitbit data and information from health surveys administered in English or Spanish were used in this study. Information about specific Fitbit tables and the survey questions used are given in Supplementary Table 4.

### Definitions for eligibility criteria

Participants met general activity eligibility criteria (*n* = 54,509) if they completed the Basics survey, had a step count >0 in the activity summary table, were ≥18 years of age at the time of their earliest activity data point, and had at least 4 days of valid activity data, where a ‘valid activity day’ is defined as having ≥10 h of data per day, and ≥100 steps but <100,000 steps in a day (Supplementary Fig. 3 and Supplementary Table 5).

Participants met seasonal activity eligibility criteria (*n* = 53,295) if they met the general activity eligibility criteria above and had at least 7 ‘valid activity days’ per month for any month during which they donated Fitbit data (Supplementary Fig. 3 and Supplementary Table 6).

Participants met general sleep eligibility criteria (*n* = 34,378) if they completed the Basics survey, were ≥18 years of age at the time of their earliest sleep data point, and had at least 4 days of ‘valid sleep data’, where valid sleep data is defined as having slept ≥4 h on at least 70% of donated days^54,55^ (Supplementary Fig. 4 and Supplementary Table 7).

Participants met seasonal sleep eligibility criteria (*n* = 33,471) if they met the general sleep eligibility criteria above and had 7 or more ‘valid sleep days’ per month for any month during which they donated Fitbit data. (Supplementary Fig. 4 and Supplementary Table 8).

Participants met the lower limb fracture case study eligibility criteria (*n* = 61) if they had both EHR records for the ‘Fracture of Lower Limb’ SNOMED concept ID 4187096 (Supplementary Table 2 for participant counts and subcodes), had Fracture of Lower Limb EHR records on at least five separate days indicating injuries serious enough to require multiple subsequent visits, and had at least 300 days of step data during the 360-day observation period (±180 days of the earliest recorded Fracture of Lower Limb EHR record). The cohort builder tool on the All of Us Researcher Workbench was used to identify the initial cohort of 2,476 participants with fracture EHR records and Fitbit data, which was then restricted based on the above criteria to a final cohort of 61 (Supplementary Fig. 5 and Supplementary Table 9).

### Enrollment and geographic analyses

Figure 1a shows cumulative start date data for all WEAR and BYOD participants (*n* = 59,018), where participants were added to the cumulative count of each cohort on the day of their earliest recorded Fitbit data. Stratification into WEAR versus BYOD cohorts is described in the section ‘Data sources and participants’. Figure 1b,c shows the state-level distributions of the WEAR and BYOD cohorts, by using each individual’s state of residence data.

### Activity analyses

Participants who met the general activity eligibility criteria (*n* = 54,509) were included in a detailed demographic analysis (Table 1) and an analysis of baseline activity levels (Table 2). Median daily steps and IQRs were calculated using all available data from the entire observation window (3 October 2009 to 30 September 2023) (Table 2). The amount and duration of valid sleep data donated by each participant varied (Supplementary Fig. 6 and Supplementary Table 5).

For the seasonal activity analysis, we first calculated the median daily step count per month for each eligible participant (*n* = 53,295). We then computed the overall monthly medians and IQRs across all participants for each month. Normalization was performed by dividing each individual’s monthly median step count by their overall monthly median step count during the entire observation window (3 October 2009 to 30 September 2023). Although data are available for the whole of this date range, only data from 1 January 2018 to 30 September 2023 are shown in Fig. 2. This date range was selected to allow visualization of seasonal oscillations and to include the COVID-19 pandemic period, during which deviations from typical seasonal trends occurred.

### Sleep analyses

Participants who met general sleep eligibility criteria (*n* = 34,378) were included in an analysis of baseline sleep duration (Table 2). Median daily main sleep durations and IQRs were computed using all available data, which ranged from 6 October 2009 and 30 September 2023. The amount and duration of valid sleep data donated by each participant varied (Supplementary Fig. 7 and Supplementary Table 7). Fitbit devices record all sleep events, differentiating between shorter periods of sleep, such as naps, and the longest sleep period, which is designated the ‘main sleep’. Next, using each participant’s median daily sleep duration, we calculated the percentage of the general sleep cohort that fell into the following categories^11^: normal sleep (7–8.99 h per night), very short sleep (<5 h per night), short sleep (5–6.99 h per night) and long sleep (≥9 h per night).

For the seasonal sleep analysis, we first calculated the median daily sleep duration per month for each eligible participant (*n* = 33,471). We then computed the overall monthly medians and IQRs across all participants for each month. Normalization for the seasonal data was completed by dividing each individual’s monthly median sleep duration by their overall median sleep duration during the entire observation window from 6 October 2009 and 30 September 2023. Although data are available for the entire observation window, only data from 1 January 2018 to 30 September 2023 are shown in Fig. 2. This subset of the data was selected for the same reasons as described above in the ‘Activity analyses’ section.

### Data type overlap analysis

All participants who donated any Fitbit data (*n* = 59,018) were assigned a flag for each data type of interest. The authors selected the highlighted data types based on their expectations of those with the broadest interest. The counts and overlap of participants donating each data type were visualized in a Venn diagram (Fig. 4). Supplementary Table 4 specifies the source data used to determine the number of participants who donated key data types in addition to any Fitbit data for this overlap analysis.

### Lower limb fracture: case study analysis

Participants who met the lower limb fracture case study eligibility criteria (*n* = 61) were included in an analysis to track the decline and recovery in median daily steps around the time of an EHR event that indicated a broken leg (see Supplementary Fig. 5 and Supplementary Tables 2 and 9 for SNOMED subcodes and participant counts). For this analysis, we deviated from our standard practice of removing days with step counts <100 or >100,000 as ‘invalid’ (see the ‘valid activity day’ definition in the ‘Activity analyses’ section). Given the nature of the analysis, we were specifically interested in anomalously low step days and the highest value seen in this cohort was 51,101 steps. As a result, no step days were excluded before analysis. To compare all participants on a uniform scale, daily step data were normalized by dividing each participant’s daily step count by their mean step count across all the days in the 360-day observation period. Plots show 30-day rolling averages of this normalized step count.

### Device type analysis

There is significant heterogeneity in the devices used in the All of Us Fitbit dataset. Although we did not account for this heterogeneity directly in our methods, we did develop a detailed catalog of all Fitbit device models present in the dataset, including participant counts for each model, sensor specifications, estimated release years and device-specific considerations (Supplementary Table 3). We also analyzed the distribution of device count per participant (Supplementary Fig. 1). Together, these resources enable researchers to understand the range of devices used and their measurement capabilities for future research.

### Statistical analyses

R and Python programming languages were used to conduct all the analyses on the All of Us Researcher Workbench. Summary demographic information is reported as count (*n*) and percentage for each cohort (WEAR, BYOD, total cohort). Summary data are reported as medians and IQRs, because most of the step and sleep data distributions are not normally distributed (either skewed, or even bimodal if zero values are included), and mean and standard deviation combinations would be inaccurate as to the actual distribution shapes. Owing to our large *N*, normality tests (Anderson–Darling, Shapiro–Wilk) typically fail, so Q–Q plots were interpreted with consistent skewing indicated. Many subsets also contained a significant portion of ‘zeros’, rendering the data bimodal. Participant counts <20 for any reporting category were obscured to protect the privacy of participants and in accordance with the All of Us Research Program’s Data and Statistics Dissemination Policy (https://www.researchallofus.org/faq/data-and-statistics-dissemination-policy).

### Reporting summary

Further information on research design is available in the Nature Portfolio Reporting Summary linked to this article.

## Online content

Any methods, additional references, Nature Portfolio reporting summaries, source data, extended data, supplementary information, acknowledgements, peer review information; details of author contributions and competing interests; and statements of data and code availability are available at 10.1038/s41591-026-04352-3.

## Supplementary information

Supplementary Information Supplementary Methods, Supplementary Tables 1–9 and Supplementary Figs. 1–7.

Reporting Summary

## Data Availability

This study used data from the All of Us Research Program’s Controlled Tier Dataset version 8 (C2024Q3R5), available to registered users on the All of Us Researcher Workbench (https://workbench.researchallofus.org). The dataset is accessible only to registered researchers to protect patient privacy. Step-by-step instructions for how an institution and individual can gain access is available at https://support.researchallofus.org/hc/en-us/articles/9005549268756-How-to-Obtain-a-DURA-with-All-of-Us.

## Code availability

Code used for this study is available to users of the All of Us Research Workbench platform in a featured workspace. In addition, a public-facing code repository (outside the Researcher Workbench) is available via GitHub at https://github.com/RTIInternational/allofus_NIH_wear.
