# Supplementary material for: The All of Us Research Program’s wearables dataset
Source: Nat Med. 2026 Apr 27;32(6):2302–10. doi: 10.1038/s41591-026-04352-3 (PMC13278962; doi:10.1038/s41591-026-04352-3)
Supplement: Supplementary file 1 — Supplementary Methods, Supplementary Tables 1–9 and Supplementary Figs. 1–7. [file 41591_2026_4352_MOESM1_ESM.pdf]

nature medicine

Supplementary information https://doi.org/10.1038/s41591-026-04352-3

The All of Us Research Program's wearables dataset

In the format provided by the authors and unedited

Supplementary Information

<u>Table of Contents</u>

- **Supplementary Methods.**
- **Supplementary Tables**
    - **Supplementary Table 1.** Basic demographics and compliance metrics of participants with any Fitbit data
    - **Supplementary Table 2.** SNOMED “Fracture of Lower Limb” concept ID 4187096 for n=61 individuals.
    - **Supplementary Table 3.** Device type information
    - **Supplementary Table 4.** *All of Us* Research Program data used in analyses.
    - **Supplementary Table 5.** Basic demographics and compliance metrics of the “General Activity Cohort
    - **Supplementary Table 6.** Basic demographics and compliance metrics of the “Seasonal Activity Cohort”
    - **Supplementary Table 7**. Basic demographics and compliance metrics of the “General Sleep Cohort
    - **Supplementary Table 8**. Basic demographics and compliance metrics of the “Seasonal Sleep Cohort”
    - **Supplementary Table 9.** Basic demographics and compliance metrics of the lower limb fracture case study cohort
- **Supplementary Figures**
    - **Supplementary Figure 1.** Absolute seasonal median daily step and sleep data
    - **Supplementary Figure 2.** Distribution of device count per participant.

- **Supplementary Figure 3.** Consort diagram showing inclusion/exclusion criteria for Fitbit activity analysis.
- **Supplementary Figure 4.** Consort diagram showing inclusion/exclusion criteria for Fitbit sleep analysis.
- **Supplementary Figure 5.** Consort diagram showing inclusion/exclusion criteria for Lower Limb Fracture analysis.
- **Supplementary Figure 6.** Device compliance metrics in participants with activity data.
- **Supplementary Figure 7.** Device compliance metrics in participants with sleep data.

This supplementary material has been provided by the authors to give readers additional information about their work.

Supplementary Methods.

Demographic and Geographic Methods

1. **Data Sources**

Person_ext table: contains “state_of_residence_source_value” data for all participants.

Basic Survey results used for demographic analysis was obtained from the ds_survey and cb_criteria tables.

Wear_study table: Used to identify participants who consented to the WEAR study.

Six data tables (activity_summary, steps_intraday, sleep_daily_summary, sleep_level, heart_rate_summary, and heart_rate_minute_level) were queried to determine the earliest fitbit data dates for each individual.

2. **Inclusion Criteria**

Fitbit Data Requirement: All individuals included in Figure 1 were required to have donated fitbit data.

WEAR cohort inclusion: Consented to the WEAR program and donated Fitbit data after 2/22/2021.

BYOD cohort inclusion: Either did not consent to inclusion in the WEAR program or began donating data before 2/22/2021.

3. **Study Period:** The data included in the analysis was available from 10/03/2009 through 10/01/2023.

4. **Data Processing and Analysis**

Statistical Analysis: Analyses in this section include stratification of participants into WEAR, BYOD, and Total cohorts, with further stratification by the demographic categories available in the Basic Survey, which include Race, Age, Sex at Birth, Income, Education, and several disability questions. Cumulative count summaries are based on the date of earliest fitbit data donation for each individual, by month. Stratification summaries in Table 1 are simple counts and percentages.

Data Extraction: SQL queries were utilized to extract relevant data including person ids, dates, activity and sleep data, survey responses, and wear study consent. Code for this extraction is located in the published github repository at: https://github.com/RTIInternational/allofus_NIH_wear

Determining BYOD versus WEAR status: WEAR status was determined by completion of the WEAR consent process using variables' resultsconsent_wear' and 'wear_consent_start_date' from the wear_study table. Participants without a WEAR consent were assigned to BYOD. In a subset of cases participants that completed a WEAR consent previously contributed Fitbit data through BYOD. These participants were reassigned to BYOD by determining the minimum date step counts were reported in the activity_summary table if this date was before the participants WEAR consent date 'wear_consent_start_date' from the wear_study table they were assigned to BYOD.

Fitbit Data Contribution Analysis: The cumulative number of participants with Fitbit activity data over time was determined based on the minimum date that either step count data was available for in the activity_summary table or the minimum date that sleep data was available in the daily_summary table.

Demographic Analysis: Demographic characteristics were extracted and analyzed from the relevant demographic tables, ensuring a comprehensive understanding of the participant cohort (Supplementary Table 1). Self-reported race/ethnicity, self-reported sex at birth, household

income, and educational attainment were collected via the Basics survey and extracted from the ds_survey table. All six disability related questions were collected via the Basics survey or the Life Functioning survey and extracted from the ds_survey table. Participant age on the date of their first step data record was calculated using their date of birth which is recorded during the *All of Us* Research Program consent process and extracted from the person table.

Activity Analysis Methods:

1. **Data Sources**

Activity Summary Table: Used for analyzing the 'steps' variable that captures the total steps taken per day for each participant.

Steps Intraday Table: Used for analyzing the 'steps' variable that captures the number of steps taken on a minute level for each participant.

Wear Study Table: Used for analyzing the 'resultsconsent_wear' and 'wear_consent_start_date' variables that capture participants who were consented for the WEAR Study.

2. **Inclusion Criteria**

Age Requirement: Participants included in the analysis were required to be 18 years or older on the date of their earliest Fitbit data record. This was to ensure that all analysis results were applicable to adult populations. For instance, if a participant's first data record was on October 10, 2009, but they were 12 years old on that date, they were excluded from all analyses.

Device Use Compliance: Participants needed to wear their Fitbit device for at least 10 hours per day for that day to be included in the analysis. This allowed for the evaluation of days with consistent Fitbit use throughout the day. The “Steps_Intraday” table provides minute level resolution of step counts throughout the day (as opposed to a single daily total step count,

available in the activity_summary table). The presence of non-zero step counts during any given hour of the 24 possible hours indicates the device is being worn, and if 10 hrs are accounted for during a daily period, this was labeled as a valid day. Less than 10 hrs of step data are considered invalid days.

Daily Step Count Requirements: Participants were required to log at least 100 steps but less than 100,000 steps per day for that day to be included in the analysis.

Minimum Days of Valid Activity Data: Each participant needed to meet the above criteria for at least four days throughout the observation period.

Seasonality Requirement: To account for seasonal variation in physical activity, participants needed to meet all general activity eligibility criteria (all 4 criteria above) for at least seven days in any given calendar month for that month of data to be included.

3. Study Period

The data included in the activity analysis was available from 10/03/2009 through 10/01/2023. The seasonality analysis data in Figure 2 was further subset to 01/01/2018 through 09/30/2023.

4. Data Processing and Analysis

Statistical Analysis: R version 4.5.0 and Python version 3.10.16 programming languages were used to conduct all the analyses on the *All of Us* Researcher Workbench. Python was used for calculation of the demographic classifications, for most table and figure generation, and for the datatype overlap analysis so that existing Python code and custom packages from the CDR v8 Data Characterization Report featured workspace[53] could be leveraged. All remaining data processing and analyses were conducted in R.

Data Extraction: SQL queries were utilized to extract relevant data including person ids, dates, step counts, and wear study consent from the activity_summary, steps_intraday, and wear_study tables to carry out activity and seasonality analysis.

Age at Earliest Fitbit Record: Age at earliest Fitbit record was determined using participants' date of birth from the person table and date of their earliest Fitbit data record. All calculated ages were floored to obtain the participant age.

Device Use and Step Count Compliance: The amount of time participants wore their Fitbit device was determined using the steps_intraday table, where the hourly step counts were checked, by creating a 'has_hour' variable that was coded 1 if greater than 0 steps were taken in the hour and 0 if no steps were taken. This variable was then summed to create a 'use compliant' variable that was coded as TRUE for 10 or more hours of step data and FALSE for days with less than 10 hours of data. The total number of steps participants took each day was determined using the activity_summary table. Participants' data for each day were only included in the analysis if the 'use compliant' variable was TRUE and they took between 100 and 99,999 steps that day. If participants who met the age requirement had less than 4 days of data meeting the Device Use and Step Count Compliance requirement, they were excluded from the general activity analyses entirely. Furthermore, if participants who met the age requirement had less than 7 days of data per month (for each month between January 2018 and September 2023) meeting the Device Use and Step Count Compliance requirement, they were excluded from the seasonality analysis entirely (see Supplementary Figure 1 for Consort Diagram).

Seasonality Analysis: For the seasonality analysis the 'steps' and 'date' variables from the activity_summary table were used to calculate the cohort median and interquartile range of participants' median daily step count per month from January 2018 and September 2023. Seasons

were defined as follows: Winter: December, January, February; Spring: March, April, May; Summer: June, July, August; Fall: September, October, November.

Sleep Analyses Methods:

1. Data Sources

Sleep Daily Summary Table: Used for analyzing the 'minute_asleep' variable that captures the total duration of sleep for each participant per day. The 'is_main_sleep' variable was also used to subset the data to participants' main sleep, removing any naps.

2. Inclusion Criteria

Age Requirement: Participants included in the analysis were required to be 18 years or older on the date of their earliest sleep record. This was to ensure that all analysis results were applicable to adult populations. For instance, if a participant's first sleep data record was on October 10, 2009, but they were 12 years old on that date, they were excluded from all analyses.

Main Sleep: Only the main sleep period of each day was considered for analysis to ensure consistency and relevance in sleep duration assessment (i.e., is_main_sleep = true from sleep_daily_summary table).

Minimum Sleep Duration: Participants needed to have at least four hours of sleep on 70% or more of the days they contributed data. This criterion was set as a quality control measure, since sleeping less than four hours for 30% or more days has previously been reported to be physiologically not sustainable.[54]

Minimum Days of Valid Sleep Data: Each participant needed to meet the above criteria for at least four days throughout the observation period.

Seasonality Requirement: To account for seasonal variation in sleep behavior, participants needed to meet all general sleep eligibility criteria (all 4 criteria above) for at least seven days in any given calendar month for that month of data to be included.

3. **Study Period**: The data include sleep records from 10/06/2009 through 09/30/2023. The seasonality analysis data in Figure 2 was further subset to 01/01/2018 through 09/30/2023.

4. **Data Processing and Analysis**

Statistical Analysis: All data processing and analysis were conducted in R version 4.5.0 and Python version 3.10.16.

Data Extraction: SQL queries were utilized to extract daily sleep durations in minutes 'minute_asleep' variable for participants' main sleep 'is_main_sleep' variable from the sleep_daily_summary table.

Age at Earliest Fitbit Record: Age at the earliest Fitbit data recorded was calculated using participants' date of birth from the person table and date of their earliest Fitbit data record. All calculated ages were floored to obtain the participant age.

Minimum Sleep Duration: The number of days each person slept less than 4 hours was determined using the 'minute_alseep' and 'is_main_sleep' variables from the sleep_daily_summary table. The number of days with a sleep duration of less than 4 hours was divided by the total number of days contributed by each participant. Participants that slept for less than 4 hours on 30% or more days were removed from analysis.

Sleep Analysis: Participants median sleep duration from 10/01/2022 through 09/30/2023 was determined by taking the median of the 'minute_asleep' variable for sleeps indicated as 'is_main_sleep' from the sleep_daily_summary table for each participant. The cohort median

sleep duration and interquartile range was determined by taking the median and interquartile range of participant median sleep durations. Participants were grouped into one of four sleep groups based on their median sleep duration. Participants were assigned to the "Very short sleep" group if they had a median sleep duration of less than 5 hours. Participants with a median sleep duration of 5 or more but less than 7 hours of sleep were assigned to the "Short sleep" group. Those with a median sleep duration of 7 or more but less than 9 hours of sleep were assigned to the "Normal sleep" group while those with median sleep duration of 9 or more hours of sleep were assigned to the "Long sleep" group

Device Type Analysis Methods

1. **Data Sources**

   Device Table: Used for determining device models used by the cohort to better describe device heterogeneity within the cohort.

2. **Inclusion Criteria**

   Device Type table: Individuals were required to have device type data listed in the Device table.

   Has Fitbit Data: Individuals were required to have the "has_fitbit" data flag present in the Person table.

3. **Study Period:** Device type data timing ranged from 01/14/2012 to 10/01/2023.

4. **Data Processing and Analysis**

   Data Extraction: SQL queries were utilized to extract person_id and device_version information from the Device table. Device type summaries are person_id level counts, meaning that presence

of each unique device_version value was checked using group_by statements on person_id or device_version.

Software and Packages used in Analyses

- **R Version 4.5.0,** Including R packages:

  bigrquery Version 1.5.1

  Hmisc Version 5.1.3

  tidyverse Version 2.0.0

  dplyr Version 1.1.4

  stringr Version 1.5.1

  data.table Version 1.15.4

  readr Version 2.1.5

  scales Version 1.3.0

  ggplot2 Version 3.5.2

  ggbeeswarm Version 0.7.2

  plotrix Version 3.8-4

  table1 Version 1.5.1

  IRdisplay Version 1.1

  htmltools Version 0.5.8.1

  tidyr Version 1.3.1

  knitr Version 1.50

  gt Version 1.1.0

  nortest Version 1.0-4

- **Python Version 3.10.16,** Including Python packages:

  pandas Version 2.0.3

pandas_gbq Version 0.17.9
numpy Version 1.24.4
scipy Version 1.11.4
tslearn Version 0.7.0
scikit-learn Version 1.6.0
Matplotlib Version 3.7.3
seaborn 0.12.2
choreographer Version 1.2.1
et_xmlfile Version 2.0.0
graphviz Version 0.21
iniconfig Version 2.3.0
kaleido Version 1.2.0
logistro Version 2.0.1
openpyxl Version 3.1.5
orjson Version 3.11.5
packaging Version 25.0
plotly Version 6.5.1
pytest Version 9.0.2
pytest-timeout Version 2.4.0
simplejson Version 3.20.2
tslearn Version 0.7.0
venn Version 0.1.3
google-cloud-bigquery Version 2.34.4
requests Version 2.32.3
IPython Version 8.21.0

Supplementary Tables

Supplementary Table 1. Basic demographics and compliance metrics of participants with any Fitbit data

	**Cohort Size (n)**	**Median (IQR) daily step count***	**Median (IQR) number of valid activity days**	**Median (IQR) wear-time per day**	**Median (IQR) length of data donation window in days**	**Median age at first fitbit submission (IQR)**	**% female (n)**
**Participants with step data present***	58,575	5,416.5 (2,887-8,008)	351 (91-975)	16.0 (13.0-18.0)	512 (110-1,598)	51 (36-64)	68.2% (39,936)
	**Cohort Size (n)**	**Median (IQR) daily sleep duration**	**Median (IQR) number of valid sleep days**	**Median (IQR) hours of device wear-time**	**Median (IQR) length of data donation window in days**	**Median age at first fitbit submission (IQR)**	**% female**
**Participants with sleep data present^**	54,313	6.16 (2.38 - 6.98)	159 (59 - 729)	N/A[ϕ]	464 (100 - 1569)	51 (37-64)	68.2% (37, 048)

*Note: While 59,018 participants have any Fitbit data (has_fitbit=1), only 58,575 have step data present. In addition, the median reported here includes reported step-counts of zero, whereas future cohorts filter out zero counts.

^While 59,018 participants have any Fitbit data (has_fitbit=1), only 54,313 have any valid sleep data.

ϕ Daily wear time not reported for participants with sleep data because this is not relevant to the valid sleep day criteria.

Supplementary Table 2. SNOMED “Fracture of Lower Limb” concept ID 4187096 conditions found for n=61 individuals.

**condition_concept_id**	**standard_concept_name**
4289914	Closed fracture of fifth metatarsal bone
4211657	Closed fracture of lower leg
441155	Closed trimalleolar fracture
40492392	Closed fracture of fibula
80232	Closed fracture of calcaneus
440238	Closed fracture of metatarsal bone
45766974	Pathologic fracture of femur at site of neoplasm
435644	Metatarsal bone fracture
4012455	Closed fracture distal tibia

condition_concept_id	standard_concept_name
438005	Closed fracture of shaft of fibula
438879	Closed bimalleolar fracture
4008706	Closed fracture of third metatarsal bone
4175171	Pathological fracture of foot
4206872	Closed fracture of great toe
75095	Closed fracture of ankle
4263360	Fracture of pubis
4134334	Fracture of distal end of fibula
40482293	Stress fracture of tibia

condition_concept_id	standard_concept_name
40491340	Closed fracture of tibia
45766819	Stress fracture of foot
45766941	Closed fracture of proximal phalanx of great toe
4136842	Fracture of cuboid
4012588	Closed fracture proximal phalanx, toe
4117116	Fracture of superior pubic ramus
4016109	Closed fracture proximal tibia, bicondylar
4015991	Closed fracture proximal tibia, medial condyle (plateau)
4015506	Closed fracture distal femur, medial condyle

condition_concept_id	standard_concept_name
760232	Closed trimalleolar fracture of left ankle
441980	Closed fracture of foot
441428	Closed fracture of lateral malleolus
436539	Closed fracture of patella
435666	Closed fracture of upper end of tibia
434500	Closed fracture of neck of femur
433612	Closed fracture of upper end of fibula
81707	Closed fracture of cuboid bone of foot
80241	Closed fracture of navicular bone of foot

condition_concept_id	standard_concept_name
45771403	Stress fracture of femur

Supplementary Table 3. Device type information

Device model name	Est. release year *	Count of participants with device models (n=52, 860 participants with device type data)	List of sensors/motors	Notes/Considerations**
Ace	2018	<20	1. MEMS 3-axis accelerometer	Device is designed specifically for children
Ace 2	2019	<20	1. MEMS 3-axis accelerometer	Device is designed specifically for children
Ace 3	2021	<20	1. MEMS 3-axis accelerometer<br>2. Vibration motor	Device is designed specifically for children
Alta	2018	797	1. A MEMS 3-axis accelerometer	
Alta HR	2018	1,271	1. A MEMS 3-axis accelerometer<br>2. Optical heart rate tracker	

Aria	Not listed in user manual	1,580	1. Load cells<br>2. Indium tin oxide (ITO) electrodes	Wi-Fi Smart Scale
Aria 2	2018	848	1. Load cells<br>2. Indium tin oxide (ITO) electrodes	Wi-Fi Smart Scale
Aria Air	2019	1,366	1. Load cells	Wi-Fi Smart Scale
Blaze	2018	726	1. MEMS 3-axis accelerometer<br>2. Altimeter<br>3. Optical heart rate tracker<br>4. Ambient light sensor	Has GPS functionality, which relies on sensors on nearby phone to capture GPS data
Charge	Not listed in user manual	285	1. MEMS 3-axis accelerometer<br>2. Altimeter<br>3. Vibration motor	
Charge 2	2018	3,263	1. MEMS 3-axis accelerometer<br>2. Altimeter<br>3. Optical heart rate tracker	Has GPS functionality, which relies on sensors on nearby phone to capture GPS data
Charge 3	2020	1,704	1. MEMS 3-axis accelerometer<br>2. Altimeter<br>3. Optical heart-rate tracker	Has GPS functionality, which relies on sensors on nearby phone to capture GPS data

			4. Vibration motor	
Charge 4	2020	3,276	1. MEMS 3-axis accelerometer<br>2. Altimeter<br>3. GPS receiver<br>4. Optical heart-rate tracker<br>5. Vibration motor	
Charge 5	2021	17,522	1. 3-axis accelerometer<br>2. Built-in GPS receiver + GLONASS<br>3. Optical heart-rate tracker<br>4. Multipurpose electrical sensors compatible with the EDA Scan app<br>5. Ambient light sensor<br>6. Vibration motor	Manual did not specify if accelerometer was "MEMS"
Charge HR	Not listed in user manual	941	1. MEMS 3-axis accelerometer<br>2. Altimeter<br>3. Vibration motor	

			4. Optical heart rate tracker	
Classic	Information not found	<20	Information not found	Could not locate online user manual<br><br>Clips onto clothing
Eos	Information not found	<20	Information not found	Reporting suggests that Eos may refer to a google internal designation created during development of the Pixel watch: https://9to5google.com/2023/06/13/google-pixel-watch-2-eos-codename/
Flex	Not listed in user manual	912	1. MEMS 3-axis accelerometer<br>2. Vibration motor	
Flex 2	2018	581	1. MEMS 3-axis accelerometer	
Force	Not listed in user manual	45	1. MEMS 3-axis accelerometer<br>2. Altimeter<br>3. Vibration motor	The Fitbit force was recalled on March 12, 2014 due to allergic reactions from materials, which caused skin irritation (https://www.cpsc.gov/Recalls/2014/Fitbit-Recalls-Force-Activity-Tracking-Wristband)
Google Pixel Watch	Not listed on device	529	1. GPS (GLONASS, BeiDou, Galileo)<br>2. Compass	

	specifications webpage		3. Altimeter<br>4. Blood oxygen sensor<br>5. Multipurpose electrical sensor<br>6. Optical heart rate sensor<br>7. Accelerometer<br>8. Gyroscope<br>9. Ambient light sensor	
Google Pixel Watch 2	Not listed on device specifications webpage	<20	1. GPS (GLONASS, BeiDou, Galileo, Quasi-Zenith Satellite)<br>2. Compass<br>3. Altimeter<br>4. Red and infrared sensors for oxygen saturation (SpO2) monitoring<br>5. Multipurpose electrical sensors compatible with the ECG app<br>6. Multi-path optical heart rate sensor<br>7. Three-axis accelerometer	

			8. Gyroscope<br>9. Ambient light sensor<br>10. Electrical sensor to measure skin conductance (cEDA) for body response tracking<br>11. Skin temperature sensor<br>12. Barometer<br>13. Magnetometer	
Inspire	2020	530	1. A MEMS 3-axis accelerometer	
Inspire 2	2020	3,177	1. 3-axis accelerometer<br>2. Multi-path optical heart-rate tracker<br>3. Vibration motor	Manual did not specify if accelerometer was "MEMS"<br><br>Has GPS functionality, which relies on sensors on nearby phone to capture GPS data
Inspire 3	2024	846	1. Optical heart-rate tracker<br>2. Red and infrared sensors for oxygen saturation (SpO2) monitoring<br>3. 3-axis accelerometer, which tracks motion patterns	Manual did not specify if accelerometer was "MEMS"<br><br>Has GPS functionality, which relies on sensors on nearby phone to capture GPS data

			4. Ambient light sensor<br>5. Vibration motor	
Inspire HR	2019	1,284	1. MEMS 3-axis accelerometer<br>2. Optical heart-rate tracker	Has GPS functionality, which relies on sensors on nearby phone to capture GPS data
Ionic	2020	264	1. MEMS 3-axis accelerometer<br>2. Altimeter<br>3. GPS receiver with GLONASS<br>4. Optical heart-rate tracker<br>5. Ambient light sensor<br>6. Vibration motor	
Luxe	2021	1,533	1. 3-axis accelerometer<br>2. Optical heart-rate tracker<br>3. Ambient light sensor<br>4. Vibration motor	Has GPS functionality, which relies on sensors on nearby phone to capture GPS data
MobileTrack	Information not found	10,177	n/a	Phone app (See related Fitbit blog post: https://community.fitbit.com/t5/Android-App/What-is-Mobile-Track/td-p/4697385)
One	Not listed in user manual	634	Not listed in user manual	Clips onto clothing

R11	Information not found	<20	Information not found	Reporting suggests that R11 may refer to a google internal designation created during development of the Pixel watch: https://9to5google.com/2023/06/13/google-pixel-watch-2-eos-codename/
Sense	2023	2,729	1. Multi-path optical heart rate tracker<br>2. Multipurpose electrical sensors compatible with the ECG app and EDA Scan app<br>3. Gyroscope<br>4. Altimeter<br>5. 3-axis accelerometer<br>6. On-wrist skin temperature sensor<br>7. Ambient light sensor<br>8. Built-in GPS receiver + GLONASS<br>9. Vibration motor<br>10. Speaker<br>11. Microphone	

Sense 2	2024	822	1. Multi-path optical heart rate tracker<br>2. Electrical sensor to measure skin conductance (cEDA)<br>3. Multipurpose electrical sensors compatible with the ECG app and EDA Scan app<br>4. Red and infrared sensors for oxygen saturation (SpO2) monitoring<br>5. Gyroscope<br>6. Altimeter<br>7. 3-axis accelerometer<br>8. On-wrist skin temperature sensor<br>9. Ambient light sensor<br>10. NFC chip<br>11. Built-in GPS receiver + GLONASS<br>12. Vibration motor<br>13. Speaker	

			14. Microphone<br>15. WiFi (deactivated, cannot be turned on)	
Surge	Not listed in user manual	200	1. MEMS 3-axis accelerometer<br>2. Altimeter<br>3. GPS receiver<br>4. Bluetooth 4.0 radio transceiver<br>5. Vibration motor<br>6. Optical heart rate tracker<br>7. Ambient light sensor	
Ultra	Not listed in user manual	49	Not listed in user manual	Clips on to clothing
Versa	2020	1,302	1. MEMS 3-axis accelerometer<br>2. Altimeter<br>3. Optical heart-rate tracker<br>4. Ambient light sensor<br>5. Vibration motor	Has GPS functionality, which relies on sensors on nearby phone to capture GPS data

Versa 2	2019	3,732	1. MEMS 3-axis accelerometer<br>2. Altimeter<br>3. Optical heart-rate tracker<br>4. Ambient light sensor<br>5. Microphone<br>6. Vibration motor	Copyright date of Versa 2 predates original Versa device<br><br>Has GPS functionality, which relies on sensors on nearby phone to capture GPS data
Versa 3	2020	5,108	1. 3-axis accelerometer<br>2. Altimeter<br>3. Built-in GPS receiver + GLONASS<br>4. Multi-path optical heart-rate tracker<br>5. Device temperature sensor (skin temperature variation available through Premium only)<br>6. Ambient light sensor<br>7. Microphone<br>8. Speaker	

			9. Vibration motor	
Versa 4	2023	10,541	1. Multi-path optical heart rate tracker<br>2. Red and infrared sensors for oxygen saturation (SpO2) monitoring<br>3. Altimeter<br>4. 3-axis accelerometer<br>5. Ambient light sensor<br>6. NFC chip<br>7. Built-in GPS receiver + GLONASS<br>8. Vibration motor<br>9. Speaker<br>10. Microphone<br>11. WiFi (deactivated, cannot be turned on)	Versa 4 attempts to connect to the GPS sensors on your phone to preserve battery life. If your phone isn't nearby or moving, Versa 4 uses built-in GPS.
Versa Lite	2020	610	1. MEMS 3-axis accelerometer<br>2. Optical heart-rate tracker	Versa Lite Edition uses the GPS sensors on your nearby phone tocapture GPS data.

			3. Ambient light sensor<br>4. Vibration motor	
Zip	Not listed in user manual	560	Not listed in user manual	Clips onto clothing

*Estimated release year is based on the copyright date from the corresponding Fitbit user manuals, which may at times reflect a manual update rather than the original device release date.

**Unless specified in the notes/considerations column, device models listed are wrist-worn devices

Supplementary Table 4. ***All of Us*** **Research Program data used in analyses.** The table describes the source, table, and element of *All of Us* Research Program data used for each variable in each analysis.

Variables used for key analyses	Source	Data table	Data elements
**person_id: PRIMARY KEY**	All	All tables listed	person_id is the unique primary key to link records from all of the tables and variables below.
**Activity Analysis**			
Step counts and valid day criteria >=100 and <100,000 steps / day	Fitbit	activity_summary	steps, date
WEAR study enrollment	Fitbit	wear_study	resultsconsent_wear, wear_consent_start_date
Valid day criteria 10 hours of data	Calculated	steps_intraday	steps, datetime
**Sleep Analysis**			
Sleep duration	Fitbit	sleep_daily_summary	minute_asleep
Date of sleep	Fitbit	sleep_daily_summary	sleep_date
Main sleep	Fitbit	sleep_daily_summary	is_main_sleep
**Demographic Characteristics**			
Self-Identified Race / Ethnicity	The Basics	person_ext	self_reported_category_source_value
Age	Consent Process	person	date_of_birth
Self-reported Sex at Birth	The Basics	ds_survey	question_concept_id = 1585845
Gender Identity	The Basics	ds_survey	question_concept_id = 1585838

Variables used for key analyses	Source	Data table	Data elements
Household Annual Income	The Basics	ds_survey	question_concept_id = 1585375
Educational Attainment	The Basics	ds_survey	question_concept_id = 1585940
Healthcare Access and Utilization	Calculated	ds_survey	Complex calculation references the CDR V8 data characterization report featured workspace [13]. The following are used in the calculation: Where any of the follow answers is 'yes' for question_concept_id is 1585386 or 43530593 and answer_concept_id is 1585388 or 43528803, OR question contains "delayed medical care" and answer_concept_id is 43530010, 43530025, 43530052, or 43530110, OR question contains "can't afford" and answer_concept_id is 43530105, 43530082, 43530034, 43530028, 43530041, 43530054, 43530237, or 43530046, OR question_concept_id is 43530416, 43530417, or 43530415 and answer contains 'yes', OR question contains "delayed medical care" and answer_concept_id is 43530258, 43530254, 43530014, or 43530033 AND question_concept_id is 43529899 and answer_concept_id is 43528387, 43529239, or 43529844

**Variables used for key analyses**	**Source**	**Data table**	**Data elements**
Are you blind or do you have serious difficulty seeing, even when wearing glasses?	The Basics, Life Functioning Survey	ds_survey	question_concept_id = 903574
Are you deaf or do you have serious difficulty hearing?	The Basics, Life Functioning Survey	ds_survey	question_concept_id = 903573
Because of physical, mental, or emotional condition, do you have serious difficulty concentrating, remembering or making decisions?	The Basics, Life Functioning Survey	ds_survey	question_concept_id = 903575
Do you have serious difficulty walking or climbing stairs?	The Basics, Life Functioning Survey	ds_survey	question_concept_id = 903576
Do you have difficulty dressing or bathing?	The Basics, Life Functioning Survey	ds_survey	question_concept_id = 903577
Because of physical, mental, or emotional condition, do you have difficulty doing errands alone such as visiting the doctor's office or shopping?	The Basics, Life Functioning Survey	ds_survey	question_concept_id = 903578
**Other Data Types**			

**Variables used for key analyses**	**Source**	**Data table**	**Data elements**
Any Fitbit data	*All of Us* Metadata (Internally generated)	cb_search_person	has_fitbit
Electronic health records	*All of Us* Metadata (Internally generated)	cb_search_person	has_ehr_data
Core surveys	*All of Us* Metadata (Internally generated)	cb_search_person	has_ppi_survey_data
Physical measurements	*All of Us* Metadata (Internally generated)	cb_search_person	has_physical_measurement_data
Genomics	*All of Us* Metadata (Internally generated)	cb_search_person	has_whole_genome_variant, has_array_data
Social Determinants of Health	Calculated	ds_survey	survey = 'Social Determinants of Health'
**Device Type Analysis**			
Device Model	Fitbit	Device	device_version

Supplementary Table 5. Basic demographics and compliance metrics of the "General Activity Cohort"

Cohort Size (n)	Median (IQR) daily step count	Median (IQR) number of valid activity days	Median (IQR) wear-time per day	Median (IQR) length of data donation window in days	Median age at first fitbit submission (IQR)	% female (n)
54,509	6,454.5 (4432-8958)	366 (93-1051)	17 (14-18)	559 (111-1711)	52 (37-65)	68.2 % (37,161)

*Note the demographics of this cohort is described in greater detail in Table 1 of the manuscript.

Supplementary Table 6. Basic demographics and compliance metrics of the "Seasonal Activity Cohort"

Cohort Size (n)	Median (IQR) daily step count	Median (IQR) number of valid activity days	Median (IQR) wear-time per day	Median (IQR) length of data donation window in days	Median age at first fitbit submission (IQR)	% female (n)
53,295	6499 (4,472-9,010)	389 (95-1,084)	17 (14-18)	597 (112-1,739)	52 (37-65)	68.1% (36,309)

Supplementary Table 7. Basic demographics and compliance metrics of the “General Sleep Cohort

Cohort Size (n)	Median (IQR) daily sleep duration (hrs)	Median (IQR) number of valid sleep days	Median (IQR) length of data donation window in days	Median age at first fitbit submission (IQR)	% female (n)
34,378	6.75<br>(6.20 - 7.25)	223<br>(69 - 1053)	679<br>(101 - 2075)	51<br>(36 - 64)	67.8%<br>(23,317)

Note: daily wear time not reported for sleep cohort because this is not relevant to the valid sleep day criteria.

Supplementary Table 8. Basic demographics and compliance metrics of the "Seasonal Sleep Cohort"

Cohort Size (n)	Median (IQR) daily sleep duration	Median (IQR) number of valid sleep days	Median (IQR) length of data donation window in days	Median age at first fitbit submission (IQR)	% female (n)
33,471	6.75 (6.2 -7.27)	234 (71-1070)	589 (89 - 1946)	51 (36 - 64)	67.8% (22,688)

Note: daily wear time not reported for sleep cohort because this is not relevant to the valid sleep day criteria.

Supplementary Table 9. Basic demographics and compliance metrics of the lower limb fracture case study cohort

Cohort Size (n)	Median age at first fracture event code (IQR)	% female (n)	Median (IQR) daily step count	Median (IQR) number of valid activity days	Median (IQR) wear-time per day
61	58 (45 - 67)	80% (49)			
**180 Days Pre-Fracture Data**			8130 (5,216 – 10,912.5)	175.5 (166.75 - 179)	18.0 (16.0, 19.0)
**180 Days Post-Fracture Data**			4,944 (3,544 – 8,399)	168 (151- 179)	18.0 (16.0, 18.0)

Supplementary Figures

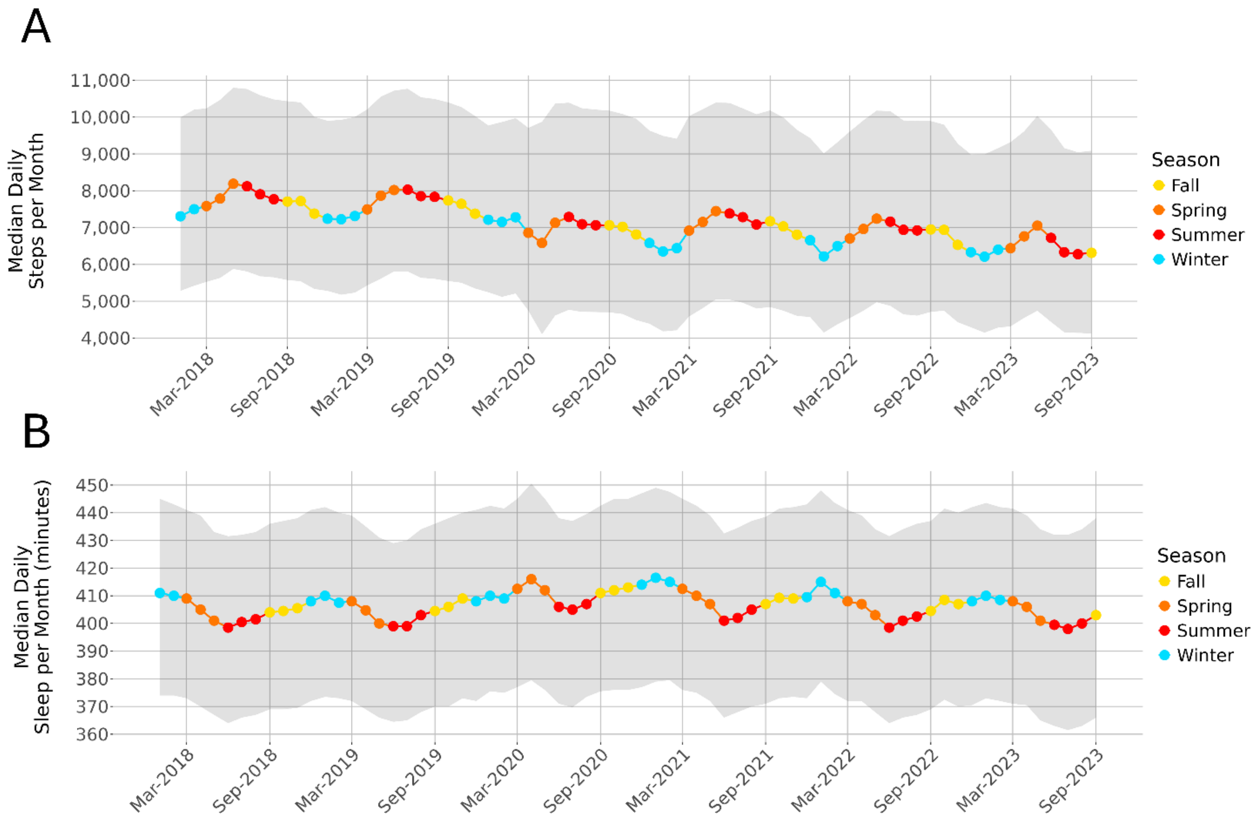

Supplementary Figure 1. Seasonal variation in absolute physical activity and sleep data. A) Seasonal variation in median daily steps of *All of Us* Research Program participants in the seasonal activity cohort (n=53,295). B) Seasonal variation in normalized median daily sleep duration of *All of Us* Research Program participants in the seasonal sleep cohort (n=33,471). For both panels, A and B, a subset of data (01/01/2018 through 09/30/2023) are shown rather than the entire sleep observation period (10/06/2009 through 09/30/2023) and the shaded area represents the interquartile range of normalized median daily steps or sleep duration for each month, respectively.

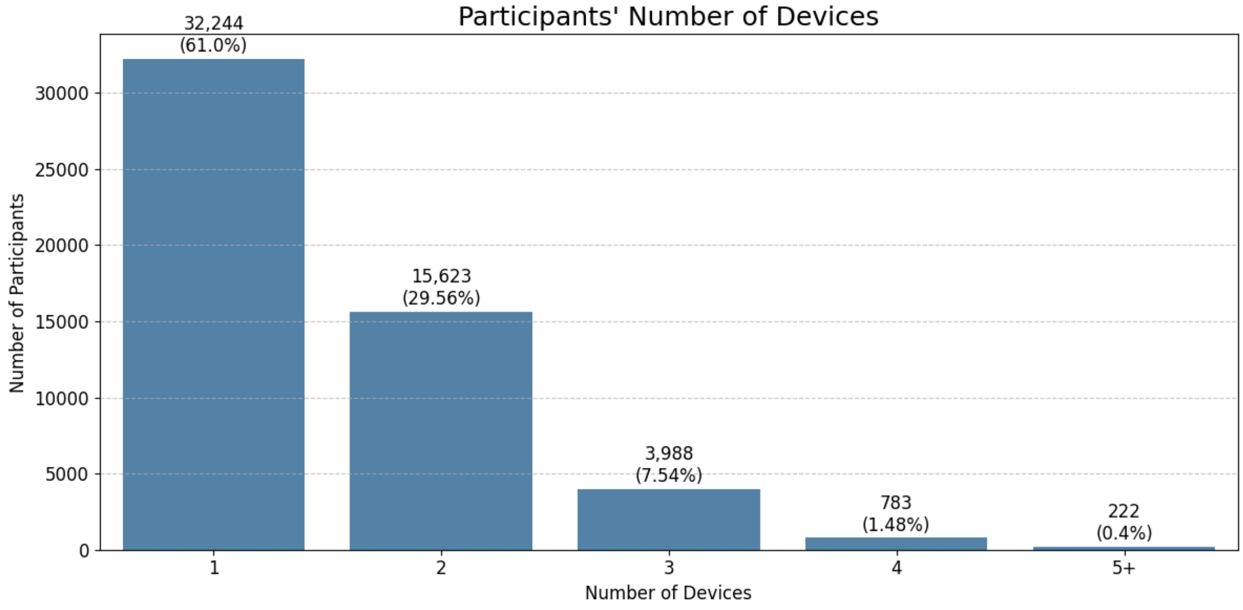

Supplementary Figure 2. Distribution of device count per participant. The bar graph displays the number and percentage of participants by device count using data available in the device table. Of the 59,018 participants with any Fitbit data (has_Fitbit=1), 52,860 (89.6%) had device data available and were represented in the device table.

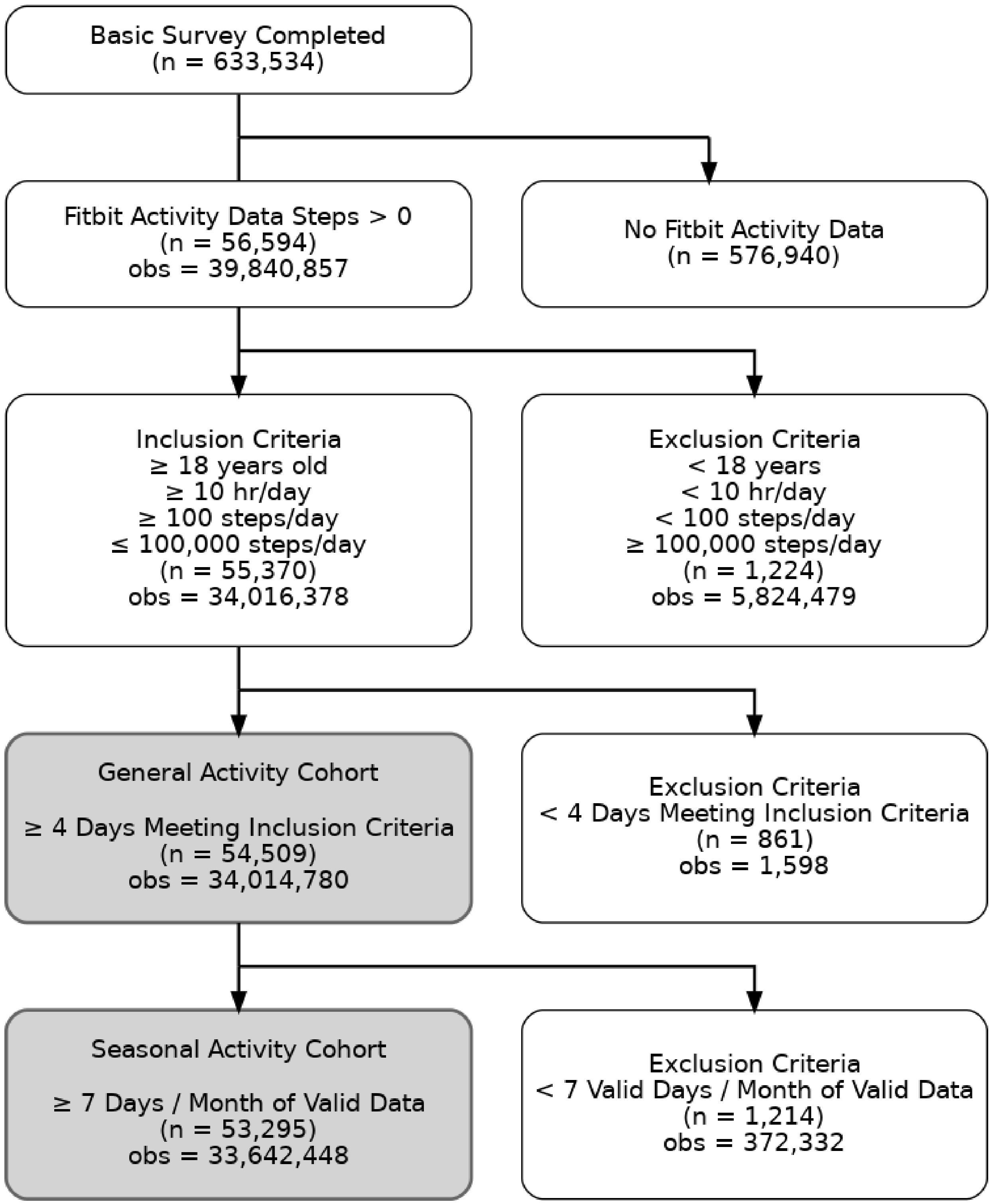

Supplementary Figure 3. Consort diagram showing exclusion criteria for Fitbit activity analysis.

The flow diagram depicts the steps utilized to produce the analytical cohort that met the exclusion criteria.

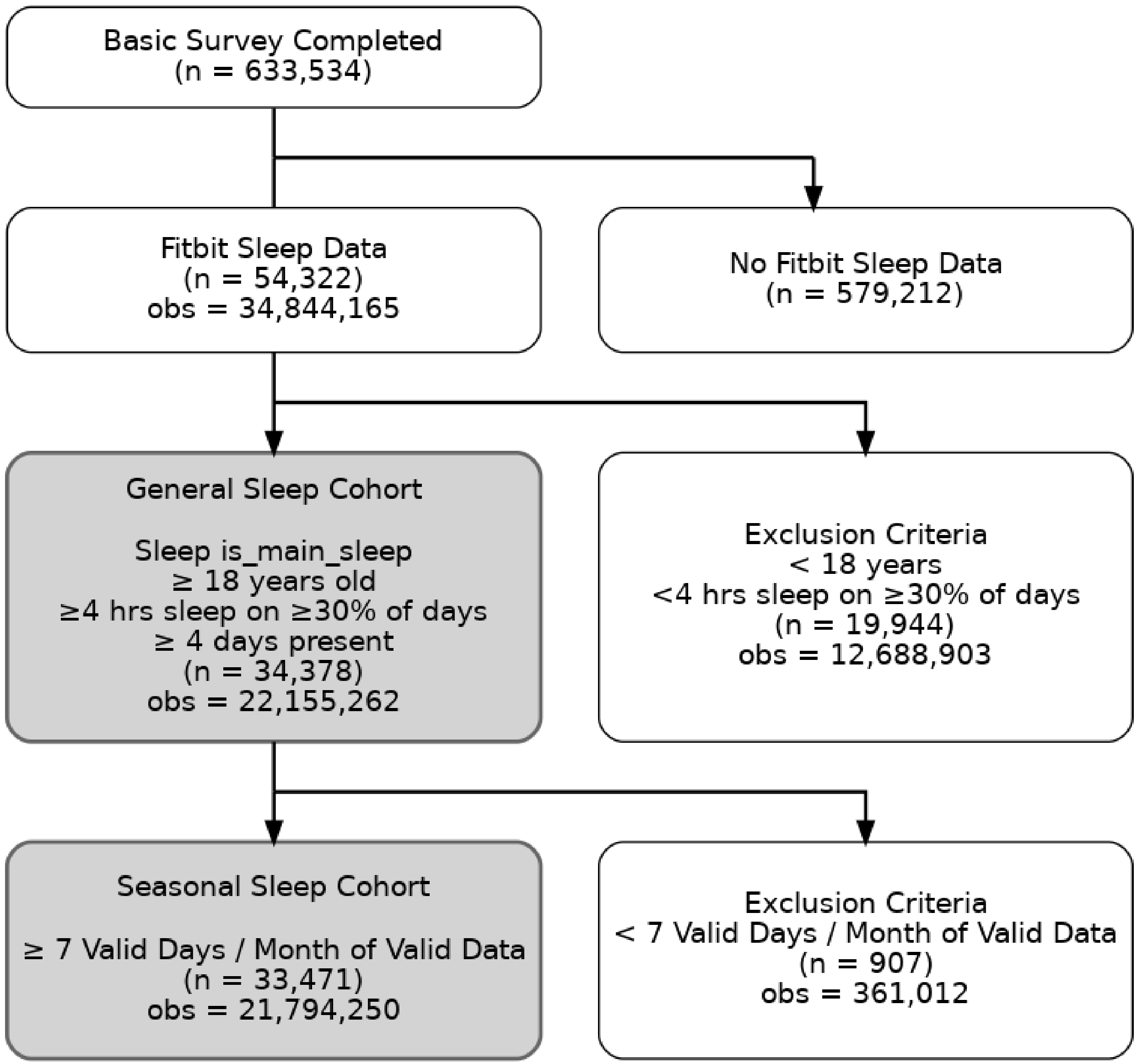

Supplementary Figure 4. Consort diagram showing exclusion criteria for Fitbit sleep analysis. The flow diagram depicts the sequential application of exclusion criteria producing the sleep eligibility group.

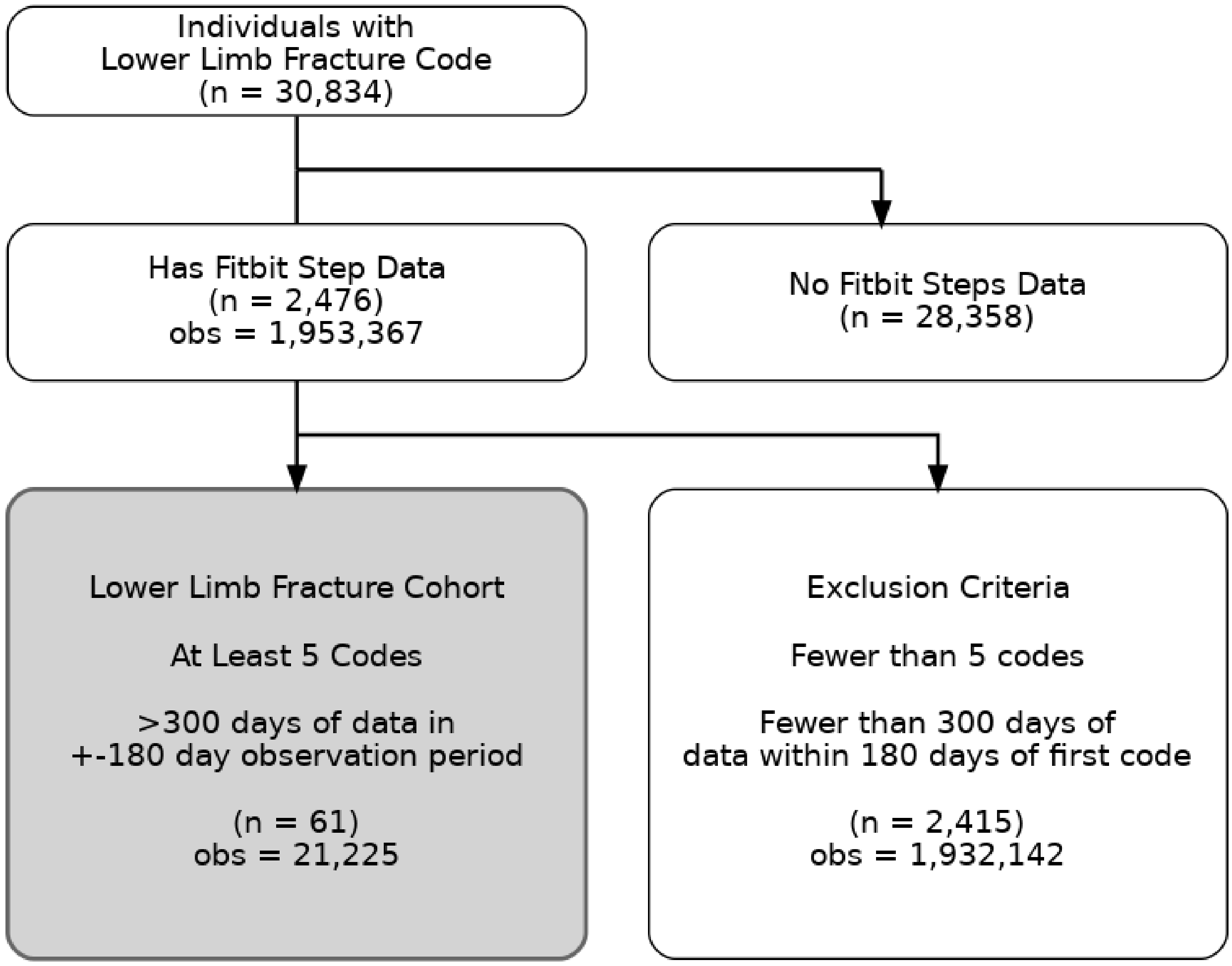

Supplementary Figure 5. Consort diagram showing exclusion criteria for Limb Fracture Cohort analysis. The flow diagram depicts the sequential application of exclusion criteria producing the final eligibility group.

Supplementary Figure 6. Device compliance metrics in participants with activity data. Histograms show multiple measures of device compliance among participants who donated activity data.

A) Valid step days per participant

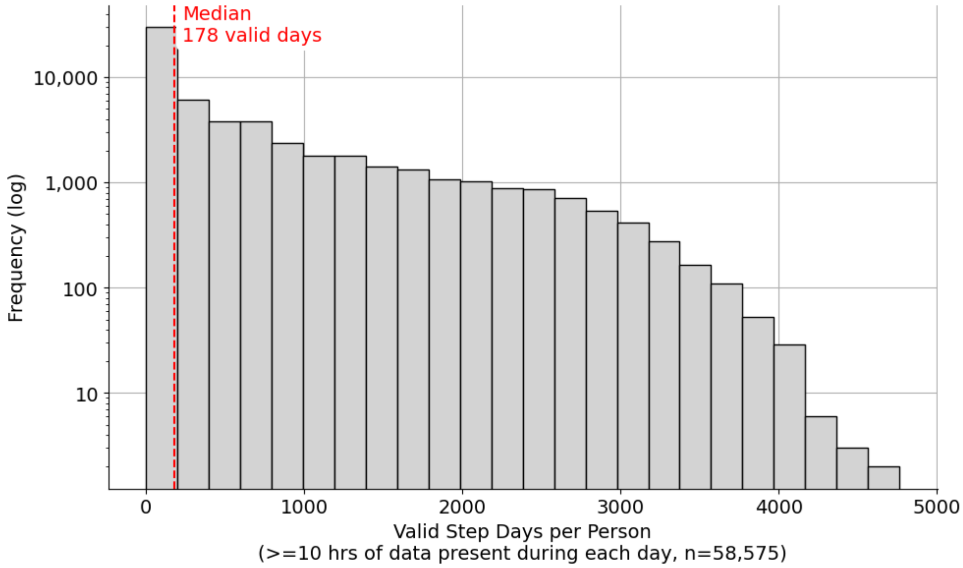

Among participants with step data (N=58,575), the median number of valid step days was 178 (IQR: 63 - 812) "valid step days". Note: While 59,018 participants have any Fitbit data (has_fitbit=1), only 58,575 have step data present; of these 3,011 participants (5.1%) had zero valid step days.

B) Step data donation window

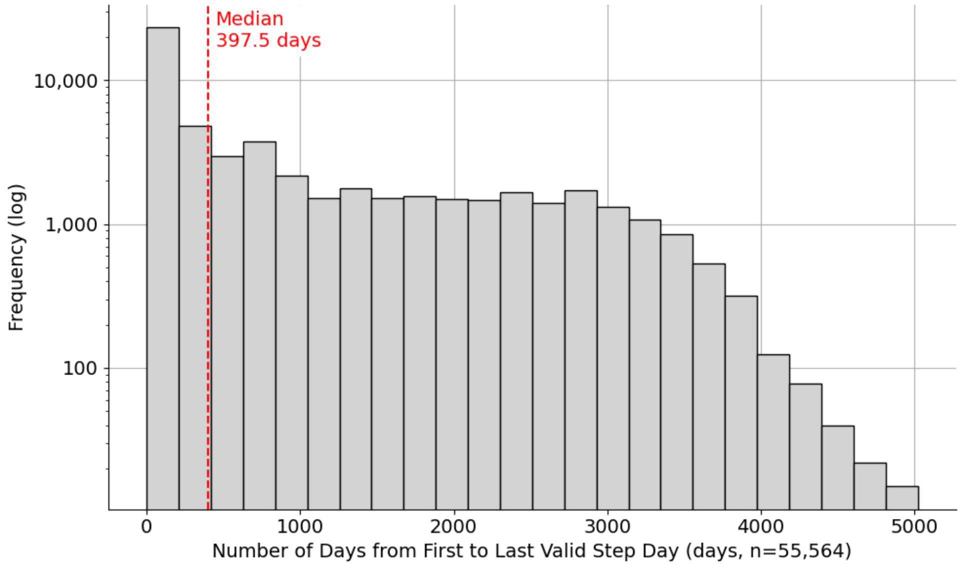

Among participants with at least one valid step day (N=55,564), the median duration from first to last valid step day was 397.5 days (IQR: 101 - 1653).

Supplementary Figure 7. Device compliance metrics in participants with sleep data. Histograms show multiple measures of device compliance among participants who donated sleep data.

A) Valid sleep days per participant

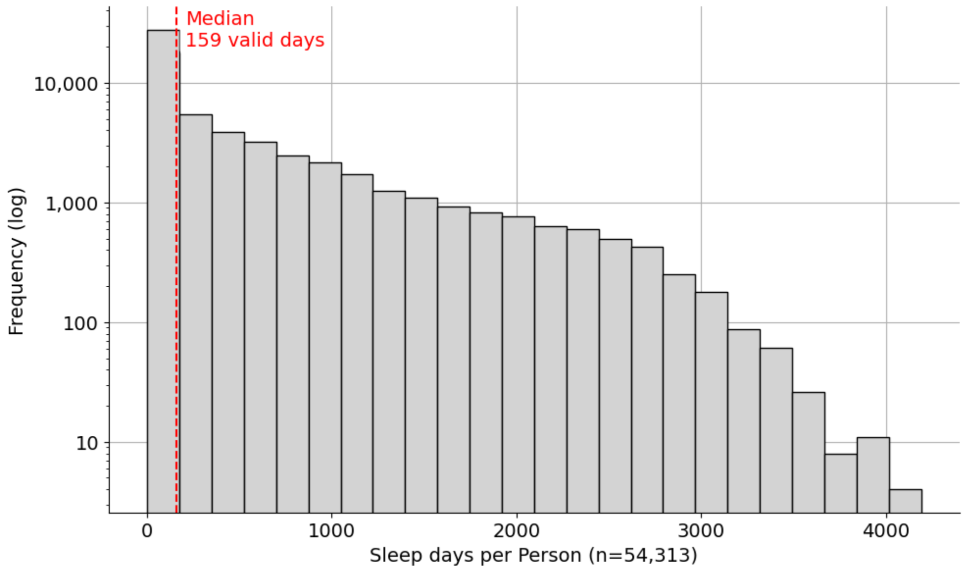

Among participants with any Fitbit data (has_fitbit=1; N=59,018), only 54,313 have "Main Sleep" data present. The median number of valid sleep days per participant was 159 (IQR: 59 - 729). Note: this data represents sleep days in the entire dataset, i.e., no sleep date restrictions were used as was done for the seasonality analysis.

B. Sleep data donation window

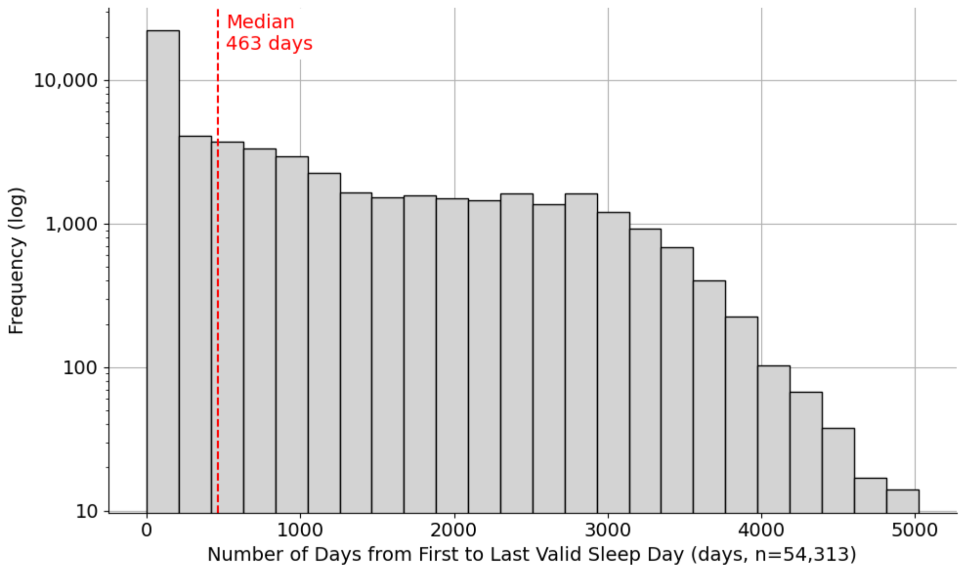

Among participants with at least one valid sleep day (N=54,313), the median duration from first to last valid sleep day was 463 days (IQR: 99 – 1,568).
