# Supplementary material for: The All of Us Research Program’s wearables dataset
Source: Nat Med. 2026 Apr 27;32(6):2302–10. doi: 10.1038/s41591-026-04352-3 (PMC13278962; doi:10.1038/s41591-026-04352-3)
Supplement: Supplementary file 2 — Reporting Summary [file 41591_2026_4352_MOESM2_ESM.pdf]

nature portfolio

Corresponding author(s): Jennifer Adjemian

Last updated by author(s): Mar 5, 2026

Reporting Summary

Nature Portfolio wishes to improve the reproducibility of the work that we publish. This form provides structure for consistency and transparency in reporting. For further information on Nature Portfolio policies, see our Editorial Policies and the Editorial Policy Checklist.

Statistics

For all statistical analyses, confirm that the following items are present in the figure legend, table legend, main text, or Methods section.

n/a	Confirmed	
☐	☒	The exact sample size (*n*) for each experimental group/condition, given as a discrete number and unit of measurement
☐	☒	A statement on whether measurements were taken from distinct samples or whether the same sample was measured repeatedly
☐	☒	The statistical test(s) used AND whether they are one- or two-sided<br>*Only common tests should be described solely by name; describe more complex techniques in the Methods section.*
☒	☐	A description of all covariates tested
☐	☒	A description of any assumptions or corrections, such as tests of normality and adjustment for multiple comparisons
☐	☒	A full description of the statistical parameters including central tendency (e.g. means) or other basic estimates (e.g. regression coefficient) AND variation (e.g. standard deviation) or associated estimates of uncertainty (e.g. confidence intervals)
☐	☒	For null hypothesis testing, the test statistic (e.g. *F*, *t*, *r*) with confidence intervals, effect sizes, degrees of freedom and *P* value noted<br>*Give P values as exact values whenever suitable.*
☒	☐	For Bayesian analysis, information on the choice of priors and Markov chain Monte Carlo settings
☒	☐	For hierarchical and complex designs, identification of the appropriate level for tests and full reporting of outcomes
☒	☐	Estimates of effect sizes (e.g. Cohen's *d*, Pearson's *r*), indicating how they were calculated

Our web collection on statistics for biologists contains articles on many of the points above.

Software and code

Policy information about availability of computer code

Data collection

The All of Us Researcher Workbench, which is a cloud computing environment that leverages Jupyter Notebooks, was used to query and analyze data from tthe All of Us Research Program's Controlled Tier Dataset version 8 (C2024Q3R5), available to registered users on the All of Us Researcher Workbench (https://workbench.researchallofus.org). The dataset is accessible only to registered researchers to protect patient privacy. Step-by-step instructions for how an institution and individual can gain access is available at this website: https://support.researchallofus.org/hc/en-us/articles/9005549268756-How-to-Obtain-a-DURA-with-All-of-Us. All code used is available at A public facing Github code repository (outside of the Researcher Workbench) is located at: https://github.com/RTIInternational/allofus_NIH_wear. For registered users of the All of Us Researcher Workbench it will also be available in a Featured Workspace at researchallofus.org.

Data analysis

The All of Us Researcher Workbench, which is a cloud computing environment that leverages Jupyter Notebooks, was used to query and analyze data from the All of Us Research Program. All code used is available at A public facing Github code repository (outside of the Researcher Workbench) is located at: https://github.com/RTIInternational/allofus_NIH_wear. For registered users of the All of Us Researcher Workbench it will also be available in a Featured Workspace at researchallofus.org. Step-by-step instructions for how an institution and individual can register is available at this website: https://support.researchallofus.org/hc/en-us/articles/9005549268756-How-to-Obtain-a-DURA-with-All-of-Us. Software and packages used in the analysis are listed below:

R Version 4.5.0, Including R packages: bigrquery Version 1.5.1, Hmisc Version 5.1.3, tidyverse Version 2.0.0, dplyr Version 1.1.4, stringr Version 1.5.1, data.table Version 1.15.4, readr Version 2.1.5, scales Version 1.3.0, ggplot2 Version 3.5.2, ggbeeswarm Version 0.7.2, plotrix Version 3.8-4, table1 Version 1.5.1, IRdisplay Version 1.1, htmltools Version 0.5.8.1, tidyr Version 1.3.1, knitr Version 1.50, gt Version 1.1.0
nortest Version 1.0-4

Python Version 3.10.16, Including Python packages: pandas Version 2.0.3, pandas_gbq Version 0.17.9, numpy Version 1.24.4, scipy Version

1.11.4, tslearn Version 0.7.0, scikit-learn Version 1.6.0, Matplotlib Version 3.7.3, seaborn 0.12.2, choreographer Version 1.2.1, et_xmlfile Version 2.0.0, graphviz Version 0.21, iniconfig Version 2.3.0, kaleido Version 1.2.0, logistro Version 2.0.1, openpyxl Version 3.1.5, orjson Version 3.11.5, packaging Version 25.0, plotly Version 6.5.1, pytest Version 9.0.2, pytest-timeout Version 2.4.0, simplejson Version 3.20.2, tslearn Version 0.7.0, venn Version 0.1.3, google-cloud-bigquery Version 2.34.4, requests Version 2.32.3, IPython Version 8.21.0

For manuscripts utilizing custom algorithms or software that are central to the research but not yet described in published literature, software must be made available to editors and reviewers. We strongly encourage code deposition in a community repository (e.g. GitHub). See the Nature Portfolio guidelines for submitting code & software for further information.

Data

Policy information about availability of data

All manuscripts must include a data availability statement. This statement should provide the following information, where applicable:

- Accession codes, unique identifiers, or web links for publicly available datasets
- A description of any restrictions on data availability
- For clinical datasets or third party data, please ensure that the statement adheres to our policy

This study used data from the All of Us Research Program's Controlled Tier Dataset version 8 (C2024Q3R5), available to registered users on the All of Us Researcher Workbench (https://workbench.researchallofus.org). The dataset is accessible only to registered researchers to protect patient privacy. Step-by-step instructions for how an institution and individual can gain access is available at this website: https://support.researchallofus.org/hc/en-us/articles/9005549268756-How-to-Obtain-a-DURA-with-All-of-Us.

Research involving human participants, their data, or biological material

Policy information about studies with human participants or human data. See also policy information about sex, gender (identity/presentation), and sexual orientation and race, ethnicity and racism.

Reporting on sex and gender	We have disaggregated data by biological sex at birth as reported in Table 2 and Supplementary Tables 5-9, with appropriate language explicitly describing this in the manuscript.
Reporting on race, ethnicity, or other socially relevant groupings	We used self-reported demographic data related to Race, Ethnicity, and American Indian and Alaska Native Status, and disability status (e.g., answers to the American Community Survey 6-question disability measure - ACS-6) in our manuscript to describe the demographics of the group of participants who donate activity Fitbit data to the All of Us Research program. We did not use these demographic variables to do any direct comparisons between groups or as proxies for other variables.
Population characteristics	See below.
Recruitment	Participants were recruited into the All of Us Research Program between May 2017 and October 2023 either at healthcare provider organization sites or directly via the enrollment website. Fitbit data were contributed through two pathways: the BYOD (Bring Your Own Device) program for participants with existing devices, or the WEAR study which provided free Fitbit devices beginning in February 2021. The cohort is older, more female, and more highly educated than the general U.S. population, especially among BYOD participants. These demographic characteristics may affect baseline activity and sleep estimates and limit generalizability of findings to the broader U.S. population.
Ethics oversight	Secondary use of All of Us Research Program data has been designated nonhuman participants research by the All of Us Institutional Review Board. Therefore, additional informed consent was not required.

Note that full information on the approval of the study protocol must also be provided in the manuscript.

Field-specific reporting

Please select the one below that is the best fit for your research. If you are not sure, read the appropriate sections before making your selection.

☐ Life sciences ☒ Behavioural & social sciences ☐ Ecological, evolutionary & environmental sciences

For a reference copy of the document with all sections, see nature.com/documents/nr-reporting-summary-flat.pdf

Behavioural & social sciences study design

All studies must disclose on these points even when the disclosure is negative.

Study description	This is a quantitative observational cohort study characterizing wearable device data from the All of Us Research Program. The study analyzes Fitbit data from 59,018 participants spanning 14 years, including over 39 million step observations and 31 million sleep observations, along with linked electronic health records and survey data. Analyses include descriptive statistics of demographic characteristics, longitudinal trends in daily steps and sleep duration, seasonal variation patterns, and a case study examining activity recovery following lower limb fractures documented in electronic health records.
Research sample	This study uses data from the All of Us Research Program (Controlled Tier CDR v8, C2024Q3R5), a National Institutes of Health initiative designed to collect health data from one million or more people living in the United States to advance precision health

research. The target enrollment of one million participants was informed by power analyses conducted by the Precision Medicine Initiative (PMI) Working Group, which concluded that a cohort of this size would provide sufficient statistical power for a broad range of precision medicine studies. The Working Group's full report and rationale are available at: https://acd.od.nih.gov/working-groups/pmi.html. The cohort represents a broad convenience sample and is not intended to be nationally representative of the U.S. population. For this study, we analyzed a subsample of 59,018 All of Us participants aged 18 years and older who contributed Fitbit wearable device data between October 2009 and October 2023. Because different research questions (e.g., seasonal trends, overall activity patterns, sleep analyses) required distinct analytic approaches, participants were grouped into five analysis cohorts with tailored inclusion and exclusion criteria to ensure data quality and validity. Cohort definitions were informed by prior research standards. The five analytic cohorts were:General activity cohort (N = 54,509; 68.2% female; median age at first Fitbit data donation = 52 years; Supplementary Table 5); Seasonal activity cohort (N = 53,295; 68.1% female; median age at first Fitbit data donation = 52 years; Supplementary Table 6); General sleep eligibility cohort (N = 34,378; 67.8% female; median age at first Fitbit data donation = 51 years; Supplementary Table 7); Seasonal sleep cohort (N = 33,471; 67.8% female; median age at first Fitbit data donation = 51 years; Supplementary Table 8); Lower limb fracture case study cohort (N = 61; 80% female; median age at first fracture EHR event code = 58 years; Supplementary Table 9).

Sampling strategy

The All of Us cohort is a broad convenience sample; therefore, findings from this study are not generalizable to the U.S. population. Participants were enrolled in the All of Us Research Program between May 2017 and October 2023 through participating healthcare provider organizations or directly via the program's online enrollment platform. The analytic sample included participants who contributed any Fitbit wearable data to the All of Us Research Program through either the Bring Your Own Device (BYOD) initiative or the WEAR program. From this group, participants were further divided into five analytic cohorts using tailored inclusion and exclusion criteria designed to ensure data quality and analytic validity for each cohort's specific research objective, consistent with prior research definitions. Detailed eligibility criteria for each cohort are provided in the Methods section. All analyses were descriptive in nature. No between-group comparisons were conducted, and covariates were not included. Because the objective was descriptive rather than inferential, no statistical methods were used to predetermine sample size. Instead, all available eligible data were included in each analytic cohort, and sample sizes reflect the total number of participants meeting criteria for that specific analysis.

Data collection

This study involved secondary use of data voluntarily shared by All of Us Research Program participants and subsequently harmonized and de-identified by the program. Since, researchers were not involved in primary data collection and blinding to experimental conditions is not applicable.

Timing

This study used the All of Us Research Program Controlled Tier CDR v8 (C2024Q3R5), which has a data cutoff date of October 1, 2023, which includes historical data extending as far back as records exist for each participant, with the earliest Fitbit data in this dataset dating to October 2009.

Data exclusions

Participants were included in analysis cohorts based on certain eligibility requirements. For example participants in the general activity cohort must have completed "The Basics" survey, been ≥18 years old at their earliest activity data point, had ≥4 days of valid activity data (defined as ≥10 hours of device wear per day with >100 and <100,000 steps), and had step counts >0 in the activity summary table. Different requirements were set for each analysis cohorts and consort diagrams are available outlining the specific exclusion criteria and counts. These exclusions were designed to ensure data quality by removing participants with insufficient device compliance, biologically implausible values, or inadequate data for the specific analyses.

Non-participation

Non-participation is not relevant to this study, which relies on secondary analysis of All of Us data.

Randomization

N/A

Reporting for specific materials, systems and methods

We require information from authors about some types of materials, experimental systems and methods used in many studies. Here, indicate whether each material, system or method listed is relevant to your study. If you are not sure if a list item applies to your research, read the appropriate section before selecting a response.

Materials & experimental systems

n/a	Involved in the study
☒	☐ Antibodies
☒	☐ Eukaryotic cell lines
☒	☐ Palaeontology and archaeology
☒	☐ Animals and other organisms
☐	☒ Clinical data
☒	☐ Dual use research of concern
☒	☐ Plants

Methods

n/a	Involved in the study
☒	☐ ChIP-seq
☒	☐ Flow cytometry
☒	☐ MRI-based neuroimaging

Clinical data

Policy information about clinical studies

All manuscripts should comply with the ICMJE guidelines for publication of clinical research and a completed CONSORT checklist must be included with all submissions.

Clinical trial registration

N/A - secondary use of de-identified clinical data

Study protocol	https://allofus.nih.gov/article/all-us-research-program-protocol
Data collection	N/A - secondary analysis of All of Us Research Program de-identified clinical data
Outcomes	Primary outcome measures were decided by the researchers, and included basic wearables outcomes, such as steps per day and duration of daily sleep. These were assessed by analyzing Fitbit data available in the All of Us Research program's Controlled Tier CDR v8 (C2024Q3R5) dataset.

Plants

Seed stocks	N/A
Novel plant genotypes	N/A
Authentication	N/A
